# Leakage-aware machine learning reveals structured clinical and vaginal microbiome patterns associated with preterm birth in a Mexican cohort

**DOI:** 10.3389/fgwh.2026.1799518

**Published:** 2026-05-07

**Authors:** Martín Ruhle, Carolina Espinosa-Maldonado, Guillermo de Anda-Jáuregui, Felipe Vadillo-Ortega, Enrique Hernández-Lemus

**Affiliations:** 1 Programa de Doctorado en Ciencias Biomédicas, Universidad Nacional Autónoma de México, Mexico City, Mexico; 2 Computational Genomics Division, Instituto Nacional de Medicina Genómica, Mexico City, Mexico; 3 Unidad de Vinculación de la Facultad de Medicina UNAM en el Instituto Nacional de Medicina Genómica, Mexico City, Mexico; 4 Programa Investigadores por México, Secretaría de Ciencia, Humanidades, Tecnología e Innovación (SECIHTI), Mexico City, Mexico; 5 Facultad de Medicina, Universidad Nacional Autónoma de México, Mexico City, Mexico

**Keywords:** cross-validation, data leakage, descriptive analytics, health equity, machine learning, Mexican population, preterm birth, vaginal microbiome

## Abstract

**Background:**

Preterm birth (PTB, <37 weeks of gestation) remains a major cause of neonatal morbidity and mortality worldwide, with Hispanic/Latino populations markedly underrepresented in microbiome-based studies, particularly in intensive data analytics scenarios.

**Methods:**

We applied leakage-aware machine learning as a descriptive analytical framework to characterize clinical and vaginal microbiome patterns associated with preterm birth in 43 pregnant Mexican women (110 longitudinal samples, 14 preterm births) recruited from public hospitals in Mexico City. Vaginal microbiome profiles (genus-level 16S rRNA V3-V4 sequencing) were analyzed using centered log-ratio transformation. We evaluated 12 model configurations representing combinations of two algorithms (Random Forest, Elastic Net), three clinical feature selection strategies (minimal DREAM-style adjustment, literature-based comprehensive features, data-driven empirical selection), and two microbiome representations (ANCOM-BC2 differentially abundant taxa, full filtered profiles). Random Forest and Elastic Net models were implemented within a rigorous subject-level nested cross-validation design to prevent data leakage. Model discrimination metrics were interpreted as indicators of internal cohort structure rather than as estimates of clinical predictive performance. Differential abundance analyses were conducted using ANCOM-BC2 both globally and within cross-validation folds to assess feature robustness.

**Results:**

The best-performing descriptive model (Random Forest with data-driven feature selection and full microbiome) exhibited AUROC 0.813±0.110, consistent with structured clinical-microbiome patterning within the cohort. Global differential abundance analysis (ANCOM-BC2, adjusted for maternal age and pre-pregnancy BMI) identified *Mycoplasma* as the only genus achieving FDR-corrected significance (LFC =+1.004, q=0.049), with ten additional genera reaching nominal significance (p<0.05). Within-fold feature importance and stability analyses consistently prioritized anthropometric variables (BMI, pre-pregnancy weight) alongside *Peptostreptococcus* and *Mycoplasma*, both detected in 100% of cross-validation iterations, indicating relative signal stability despite limited sample size.

**Conclusions:**

This study illustrates how descriptive, leakage-aware machine learning can organize, prioritize, and interpret clinical and microbiome signals in small, underrepresented cohorts. At this stage, it does not yet present a clinically deployable predictor for preterm birth, but we are working towards this definite goal in the future, with prenatal screening strategies in mind. The observed internal discrimination reflects, in this sense, cohort-specific structure rather than validated predictive performance and establishes a methodological basis for future externally validated classifiers in Latin American populations.

## 1 Introduction

Preterm birth (PTB), defined as delivery before 37 weeks of gestation, remains a leading cause of neonatal morbidity and mortality worldwide and is associated with long-term health consequences extending into childhood and adulthood [1]. Despite advances in obstetric care, the global incidence of preterm birth has not declined substantially, affecting approximately 15 million births annually, reflecting the multifactorial and incompletely understood nature of its underlying mechanisms [2]. Current clinical approaches to risk stratification, including cervical length measurement and fetal fibronectin testing, exhibit limited predictive accuracy and are not universally effective across populations [3].

Infectious and inflammatory processes have long been implicated in the pathophysiology of spontaneous preterm labor, particularly in early-onset cases [4]. Within this context, the vaginal microbiome has emerged as a biologically plausible contributor to pregnancy outcomes. Prior studies have reported associations between altered vaginal microbial community structure—characterized by reduced dominance of protective *Lactobacillus* species and increased representation of potentially pathogenic anaerobes—and increased risk of spontaneous preterm birth [5, 6]. However, vaginal microbiome composition is characterized by high inter-individual variability and strong dependence on population-specific factors including ethnicity, socioeconomic context, diet, and environmental exposures, complicating direct translation of findings across cohorts [7].

The increasing availability of high-dimensional microbiome sequencing data has motivated the application of machine learning methods to identify patterns associated with pregnancy outcomes. Supervised learning approaches have been used to classify preterm vs. term births and to prioritize candidate microbial biomarkers, including within large-scale benchmarking efforts such as the Microbiome Preterm Birth DREAM Challenge, which aggregated data from over 1,400 pregnancies across nine independent cohorts [8]. While these studies highlight the potential of computational approaches, they also underscore persistent challenges related to cohort heterogeneity, class imbalance, variable sampling protocols, and the critical risk of information leakage—particularly problematic in small or longitudinally sampled datasets where improper cross-validation can yield grossly inflated performance estimates [9].

These challenges are substantially amplified in populations that remain underrepresented in microbiome research, including Latin American cohorts. Differences in genetic ancestry, socioeconomic determinants, healthcare infrastructure, dietary patterns, and environmental exposures limit the generalizability of predictive models trained predominantly in European or North American populations. Importantly, no published machine learning studies for preterm birth prediction exist specifically for Mexican populations, representing a critical equity gap in precision medicine for pregnancy complications. As a result, there is an urgent need for analytical frameworks that prioritize methodological rigor, transparency, and interpretability when working with limited and heterogeneous datasets from underrepresented populations, rather than focusing exclusively on predictive performance metrics that may not generalize.

Recent methodological reviews have highlighted that small-sample microbiome studies frequently report inflated performance metrics due to information leakage, inadequate cross-validation strategies, and post-hoc optimization of classification thresholds on test data [9, 10]. These issues are particularly problematic when studies claim clinical utility without external validation. An alternative paradigm is to explicitly reframe machine learning as a *descriptive analytical tool* for organizing complex data and identifying stable feature associations, rather than as a predictive model ready for clinical deployment. This descriptive approach is especially appropriate for exploratory research in underrepresented populations where large validation cohorts are not yet available, allowing rigorous signal characterization while deferring claims of clinical actionability until external validation becomes feasible.

In this study, we apply leakage-aware machine learning as a descriptive analytical framework to characterize clinical and vaginal microbiome patterns associated with preterm birth in a Mexican pregnancy cohort. To our knowledge, this represents the first application of machine learning methods to vaginal microbiome data from a Mexican population. By enforcing stringent subject-level cross-validation, systematically comparing multiple feature selection strategies, and emphasizing feature recurrence and internal cohort structure over classifier optimization, we aim to identify reproducible cohort-specific signals that can inform hypothesis generation for future validation studies. Critically, we explicitly avoid strong claims of clinical deployability, instead positioning our analysis as a transparent methodological foundation for exploratory microbiome research in pregnancy complications within Latin American populations.

## 2 Materials and methods

### 2.1 Study design and participants

Following a nested case-control design with longitudinal sampling analyzed cross-sectionally, this study evaluated vaginal microbiome samples from 43 pregnant women (110 longitudinal samples). Participants were recruited between January 2022 and March 2024 at Hospitals Dr. Enrique Cabrera and Centro de Salud Jalalpa El Grande, from the Secretaría de Salud del Gobierno de la Ciudad de México, Mexico City, Mexico. The cohort was designed as a case-control enriched study including 14 preterm births (PTB, delivery <37 weeks’ gestation, representing 32.6% at subject level) and 29 term births (≥37 weeks, 67.4%). The enriched sampling design provided adequate representation of both outcome classes despite modest total sample size, enabling exploratory model development while acknowledging that clinical deployment would require recalibration to population-level PTB prevalence (approximately 10% in Mexico).

#### 2.1.1 Inclusion criteria

- Singleton pregnancies with gestational age <18 weeks at enrollment, documented by reliable last menstrual period and confirmed by first-trimester ultrasound
- Maternal age ≥18 years at enrollment
- Commitment and availability to attend all scheduled prenatal visits during pregnancy
- Availability of vaginal microbiome samples collected during pregnancy using standardized techniques
- Complete clinical metadata including anthropometric measurements, obstetric history, and pregnancy outcome ascertainment
- Provision of written informed consent

#### 2.1.2 Exclusion and elimination criteria

- Pre-existing medical complications of pregnancy prior to enrollment
- Active smoking, alcohol consumption, or illicit drug use
- Multiple gestations (twins, triplets, or higher-order pregnancies)
- Major fetal anomalies detected by prenatal ultrasound
- Insufficient DNA yield or sequencing quality for 16S rRNA profiling (library size <3,000 reads after quality filtering)
- Incomplete pregnancy outcome information
- Voluntary withdrawal from the study
- Identification of new severe medical complications strictly at the time of enrollment

Participants were classified retrospectively according to pregnancy outcome: preterm birth (delivery <37 weeks’ gestation, n=14) or term delivery (≥37 weeks, n=29). Among PTB cases, gestational ages at delivery ranged from 20.0 to 36.4 weeks (mean ± SD: 33.7±4.1 weeks). The distribution comprised: 1 very early preterm (<28 weeks), 4 moderate preterm (32–34 weeks), and 9 late preterm births (34–36 weeks).

**Figure 1:**
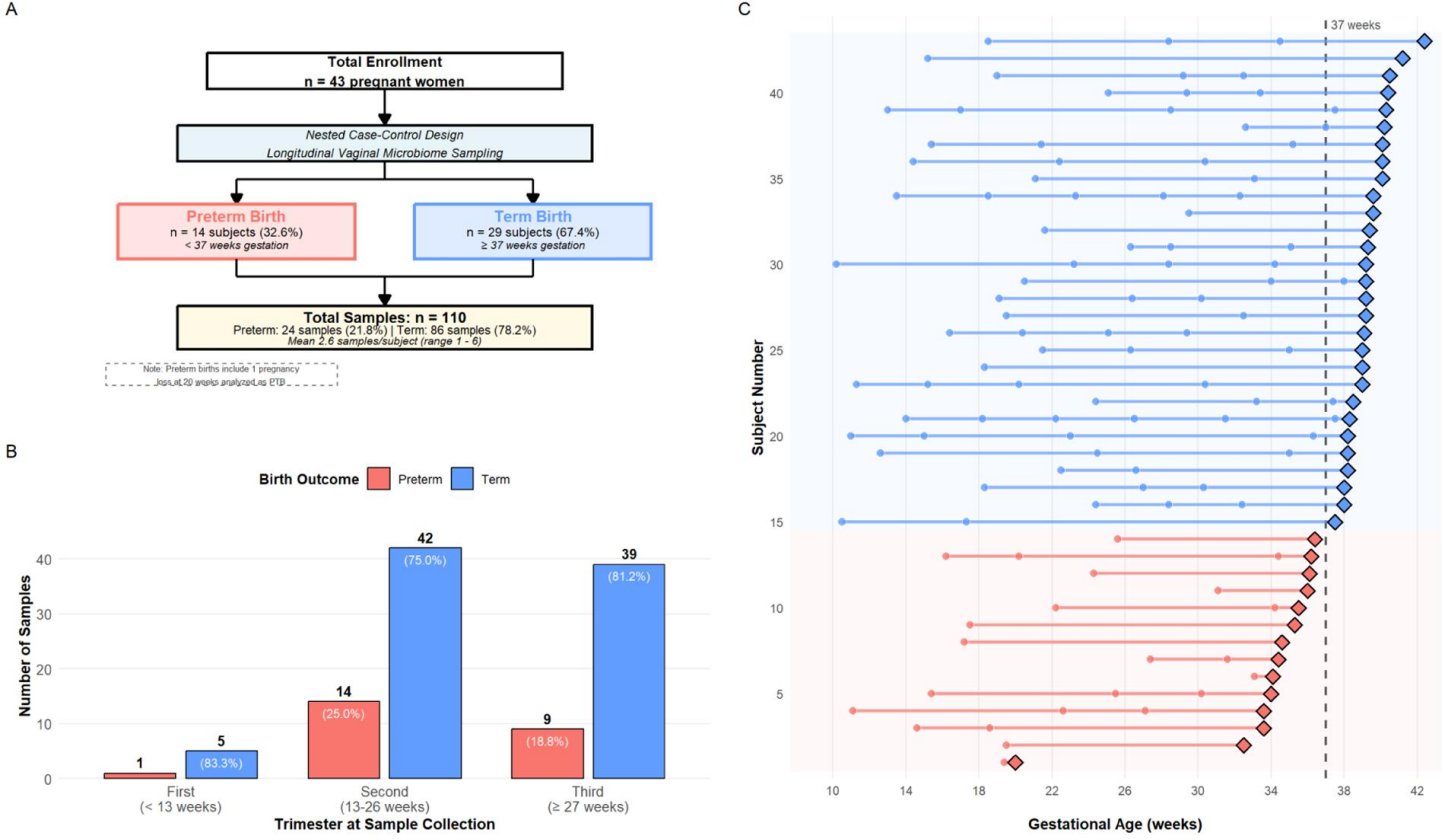
Study cohort and longitudinal sampling design. **(A)** Flowchart of nested case-control with longitudinal sampling analyzed cross-sectionally study design showing 43 participants (14 preterm, 29 term births) providing 110 vaginal microbiome samples. Inclusion and exclusion criteria detailed in Methods. **(B)** Sample distribution across pregnancy trimesters stratified by outcome. Sample collection was concentrated in first (42.7%) and second (40.0%) trimesters, with 17.3% in third trimester. PTB pregnancies contributed fewer samples (mean 1.7 samples) than term pregnancies (mean 3.0 samples) due to earlier delivery interrupting longitudinal follow-up. **(C)** Individual participant trajectories from first sample collection to delivery. Horizontal lines represent individual participants (ordered by gestational age at delivery); circles indicate timing of vaginal swab collection; diamonds mark delivery; dashed vertical line at 37 weeks delineates preterm (left) vs. term (right) deliveries. Red participants delivered preterm; blue delivered at term. Participants provided mean 2.6 samples (range 1–6) with heterogeneous timing across gestation, reflecting opportunistic sampling during routine prenatal care rather than protocol-mandated fixed timepoints.

Figure 1 provides a complete overview of participant enrollment, sample collection, and outcome classification.

One participant delivered at exactly 20.0 weeks’ gestation, which by WHO convention (≥20 weeks) classifies as preterm birth and was retained in the primary analysis. The potential influence of this case on model results is examined in the Discussion (Section 4.6).

The study was approved by the research and ethics committees of the Secretaría de Salud de la Ciudad de México (SEDESA CDMX) and the Comisión Nacional de Bioética, with registration numbers 210-101-31-17 (SEDESA CDMX), CONBIOETICA-09-CEI-004-20180213 (Comisión Nacional de Bioética), and 110-010-10-19 (SEDESA CDMX, protocol extension). All participants provided written informed consent prior to enrollment.

### 2.2 Sample collection and longitudinal sampling structure

Vaginal samples were collected longitudinally during routine prenatal visits using standardized mid-vaginal swab collection procedures. Swabs were immediately placed in cryovials and stored at $-80^{\circ }\text{C}$ until batch processing. The number of samples per participant varied between one and six (mean 2.6±1.4 samples, median 2 samples), reflecting real-world clinical follow-up patterns rather than a controlled sampling schedule with fixed gestational timepoints. This heterogeneity, while limiting temporal inference, enhances the ecological validity and generalizability of findings to clinical settings where sampling occurs opportunistically during standard prenatal care.

Longitudinal sampling throughout pregnancy yielded 110 total observations. Gestational age at sample collection ranged from 8.1 to 38.3 weeks (mean ± SD: 22.1±7.8 weeks for all samples). PTB pregnancies contributed 24 samples (21.8% of total), while term pregnancies contributed 86 samples (78.2%). Sample collection was concentrated in the first trimester (42.7% of samples) and second trimester (40.0%), with 17.3% collected in the third trimester. This distribution reflected clinical access patterns at public health facilities where women typically initiate prenatal care. The study design and longitudinal sampling structure are illustrated in Figure 1.

### 2.3 Microbiome sequencing and bioinformatics processing

#### 2.3.1 DNA extraction and 16S rRNA gene sequencing

DNA was extracted from vaginal swabs using the **NEBNext${}^{®}$ Microbiome DNA Enrichment Kit (New England Biolabs)**. The V3-V4 hypervariable region of the bacterial 16S rRNA gene was amplified using the established primer set **341F (CCTACGGGNGGCWGCAG) and 805R (GACTACHVGGGTATCTAATCC)** under the following PCR conditions: **initial denaturation at $95{\;}^{\circ }\text{C}$ for 10 min, followed by 35 cycles of $95{\;}^{\circ }\text{C}$ for 30 s, $60{\;}^{\circ }\text{C}$ for 15 s, and $72{\;}^{\circ }\text{C}$ for 50 s, with a final extension at $72{\;}^{\circ }\text{C}$ for 5 min**.

Library quantification was performed via fluorometry using the Qubit dsDNA HS Assay (Invitrogen, Cat. No. Q32851) on a Qubit 3.0/4.0 Fluorometer to ensure specific double-stranded DNA quantification. Library fragment size verification was conducted using the Agilent 4,200 TapeStation with High Sensitivity D1000 ScreenTapes (Agilent, Cat. No. 5067-5584/5585). Final library nanomolarity for clustering generation was calculated based on Qubit concentration and TapeStation average fragment size.

Sequencing was performed in a single batch to minimize batch effects, using the Illumina NextSeq 2000 platform with a P1 600-cycle flow cell generating 2×300 bp paired-end reads. The sequencing run yielded a total of 79.84 Gbp with an average quality score of 85.46% ≥ Q30.

#### 2.3.2 Bioinformatics pipeline

Raw sequence data were processed using the QIIME2 workflow (v2023.5) [11]. Quality filtering, denoising, and amplicon sequence variant (ASV) inference were performed using DADA2 with default parameters optimized for Illumina NextSeq data. Taxonomic classification was conducted using the SILVA 138 reference database with a naive Bayes classifier trained on the V3-V4 region.

#### 2.3.3 Quality control and filtering

Samples with library sizes below 3,000 reads were excluded from analysis (n=1 sample excluded). ASVs present in fewer than 5% of samples were removed to reduce spurious low-abundance features (38 low-prevalence genera excluded, retaining 59 genera for downstream analysis). After quality control, 110 samples from 43 participants were retained for analysis, yielding 59 genus-level taxa after prevalence filtering.

#### 2.3.4 Computational decontamination

To address the potential influence of reagent-derived environmental bacteria, we applied a rigorous computational decontamination strategy prior to downstream analyses. Because negative extraction controls were not sequenced in this study—a limitation of our opportunistic sampling design—we employed two complementary strategies to identify and remove putative contaminants.

First, we utilized the frequency-based method of the decontam R package [12], which identifies contaminants as taxa whose frequency correlates inversely with DNA concentration. We used post-library-prep Qubit concentrations as a proxy for sample biomass. No taxa reached statistical significance at the default threshold (p<0.10), consistent with the attenuated contaminant-concentration signal expected when using post-amplification measurements.

Second, and as our primary decontamination strategy, we compiled a literature-curated contaminant list based on genera explicitly reported in sequenced negative controls by Salter et al. [13]. This reference-based approach is conservative and anchored in established literature regarding reagent contamination. Nine genera present in our dataset matched this list and were flagged as putative contaminants: *Methylobacterium*, *Methylorubrum*, *Ralstonia*, *Mesorhizobium*, *Microbacterium*, *Bradyrhizobium*, *Sphingomonas*, *Pseudomonas*, and *Acinetobacter*. We additionally removed the non-bacterial taxon *o__Chloroplast* (plant-derived 16S reads). In total, 10 entries were excluded from the genus-level abundance table. The resulting decontaminated dataset contained 49 genera after prevalence filtering, used for all machine learning analyses, while the full decontaminated set (87 genera, without prevalence filtering) was used for global differential abundance testing via ANCOM-BC2.

### 2.4 Compositional data transformation

Given the compositional nature of microbiome count data—where relative abundances are constrained to sum to unity and interdependencies between taxa violate standard statistical assumptions—genus-level relative abundances were transformed using centered log-ratio (CLR) transformation prior to all multivariate and machine learning analyses [14, 15].

The CLR transformation maps compositional data from the constrained simplex to an unconstrained Euclidean space, enabling the use of standard statistical and machine learning methods without spurious correlations. For a composition $x=(x_{1},x_{2},\ldots ,x_{D})$ representing D taxa, the CLR transformation is defined as (Equation 1):

$$\text{CLR}(x_{i})=\ln\left(\frac{x_{i}}{g(x)}\right)$$ (1)

where $g(x)=(\prod_{\;j=1}^{D}x_{j}{)}^{1/D}$ is the geometric mean of the composition. To handle compositional zeros (taxa absent in a sample), a pseudocount was added to all relative abundances prior to CLR transformation, as recommended for microbiome compositional data [15]. A pseudocount of 0.65 was selected *a priori* as an intermediate value within the range commonly reported in the microbiome literature (0.5–1.0), and was applied uniformly across all 12 model configurations prior to any model training. To evaluate robustness to this choice, a sensitivity analysis was conducted comparing pseudocount values of 0.5, 0.65, and 1.0 across all model configurations (Supplementary Table S7). Model rankings and discrimination performance were stable across all three values, confirming that the findings reported here are not materially dependent on the specific pseudocount chosen.

Critically, CLR transformation was performed within cross-validation folds—separately for each training set—to prevent data leakage. For global analyses (e.g., principal component analysis), CLR transformation was applied to the complete dataset. For machine learning model training, CLR transformation was performed independently within each outer fold: the geometric mean was calculated from the training set only, and this training-derived geometric mean was used to transform both the training and corresponding test sets. This ensures that no information from test samples influences the transformation parameters used during model training.

### 2.5 Clinical variables and feature engineering

Clinical and anthropometric variables available for analysis included maternal demographic characteristics, pre-pregnancy and pregnancy anthropometric measurements, obstetric history, and selected pregnancy complications. The complete set of collected variables is larger and includes fetal and neonatal domains; however, this initial publication focuses on maternal clinical features most directly relevant to preterm birth risk based on established epidemiological evidence. Complete variable definitions and measurement protocols are documented in the project’s data dictionary (Supplementary Material and Data Availability section). The following clinical domains were captured:

#### 2.5.1 Demographic variables

- Maternal age (continuous, years)
- Educational attainment (categorical: primary or less, secondary, vocational/technical, associates or higher)
- Marital status (categorical)
- Employment status (binary: working outside home)

#### 2.5.2 Anthropometric variables

- Pre-pregnancy weight (continuous, kg) and height (continuous, cm)
- Pre-pregnancy body mass index (BMI, continuous, kg/m${}^{2}$)
- BMI category (categorical: underweight <18.5, normal 18.5–24.9, overweight 25.0–29.9, obese ≥30.0)
- Weight at each prenatal visit (continuous, kg)
- BMI at each visit (continuous, kg/m${}^{2}$)

#### 2.5.3 Obstetric complications and clinical events

Binary indicators (0 = absent, 1 = present) for clinically significant complications:

- First trimester bleeding
- Cervicovaginal infections
- Sexually transmitted infections
- Preeclampsia
- Preterm premature rupture of membranes (*rpm_preterm*)
- Term rupture of membranes
- Gestational diabetes mellitus
- Oligohydramnios
- Intrauterine growth restriction (IUGR)

#### 2.5.4 Hematological and nutritional variables

- Hemoglobin concentration (continuous, g/dL)
- Anemia status at any visit (binary, defined as hemoglobin <11.0 g/dL)
- Dietary supplement use (categorical)
- Vitamin supplementation (categorical)

#### 2.5.5 Temporal and sampling variables

- Gestational age at sample collection (continuous, weeks)
- Number of prenatal visits (count)

No imputation was performed for missing clinical data. Analyses were restricted to variables with sufficient completeness (>70% non-missing) across the cohort. Variables with high missingness (≥30%) were excluded from model training to prevent bias and maintain statistical power.

### 2.6 Feature selection strategies

To systematically evaluate how clinical covariate selection influences model performance and interpretability, we compared three conceptually distinct approaches to clinical feature selection. These strategies represent different philosophies in prediction modeling: minimal benchmark adjustment, evidence-based comprehensive profiling, and data-driven empirical selection tailored to the study population.

#### 2.6.1 Approach 1: minimal DREAM-style adjustment

**Rationale:** The Microbiome Preterm Birth DREAM Challenge established international benchmarks for microbiome-based PTB prediction using minimal clinical covariate adjustment—specifically, gestational age at sample collection, maternal age and maternal race/ethnicity [8]. This parsimonious approach tests whether microbiome features provide predictive value beyond basic demographic confounders and sampling timing, while maximizing comparability with published benchmarks.

**Implementation:** We included only two clinical variables:

- Gestational age at sampling (*sdg_visita*, continuous, weeks)
- Maternal age (*edad_cronologicamujer*, continuous, years; substituted for race/ethnicity as a demographic confounder, given homogeneity of the Mexican cohort)

This minimal feature set (2 clinical covariates + microbiome features + Shannon diversity) minimizes overfitting risk with small sample size.

#### 2.6.2 Approach 2: literature-based comprehensive features

**Rationale:** Extensive epidemiological literature has identified numerous clinical risk factors for preterm birth, including maternal demographics, anthropometric characteristics, obstetric history, and pregnancy complications [1]. This approach leverages established knowledge to construct evidence-based feature sets, testing whether comprehensive clinical profiling enhances microbiome-based pattern characterization.

**Implementation:** We selected 10 clinical variables with demonstrated associations to PTB in published literature, prioritizing:

1. **Direct PTB mechanisms** with relative risks >2.0: first trimester bleeding, preterm premature rupture of membranes, preeclampsia
2. **Robust demographic factors**: maternal age (U-shaped risk), pre-pregnancy BMI (U-shaped or threshold effects)
3. **Continuous physiological measurements**: hemoglobin concentration, folic acid supplementation
4. **Markers of placental/metabolic dysfunction**: oligohydramnios, gestational diabetes mellitus (though GDM more commonly associates with indicated rather than spontaneous PTB, we retained it for exploratory purposes), IUGR

The ten evidence-based variables including maternal age, pre-pregnancy BMI, hemoglobin concentration, gestational age at sampling, and binary indicators for pregnancy complications (gestational diabetes, preeclampsia, first trimester bleeding, preterm PROM, oligohydramnios, and IUGR).

#### 2.6.3 Approach 3: data-driven population-optimized features

**Rationale:** While literature-based approaches leverage global evidence, they may miss population-specific risk factors particularly relevant to Mexican pregnancies. This exploratory strategy uses data-driven univariate screening to identify the strongest predictors within this cohort, potentially uncovering locally important features not prominent in studies conducted in predominantly European or North American populations.

**Critical Methodological Note:** To prevent data leakage, univariate feature selection was performed *independently within each outer cross-validation fold* using only training subjects. This means feature sets varied across folds (which is expected and correct), and test subjects never influenced variable selection decisions.

**Implementation:**

1. **Within each outer training fold:** Univariate association testing was conducted for all available clinical variables against PTB outcome using appropriate statistical tests (*t*-test or Mann-Whitney U for continuous variables, chi-square or Fisher’s exact test for categorical variables)
2. **Ranking by strength of association:** Variables were ranked by univariate association strength (smallest p-value ranked first)
3. **Top 10 selection:** The 10 variables with strongest univariate associations in that training fold were selected, regardless of p-value threshold. This ensures consistent dimensionality (10 features) across folds while adapting to fold-specific patterns, providing dimensionality consistent with the literature-based approach.
4. **Mandatory inclusions:** Gestational age at sampling and maternal age were always included (to maintain comparability with Approaches 1 and 2), replacing lower-ranked features if not selected by univariate screening

This empirical approach allows population-specific risk patterns to guide feature selection while maintaining methodological rigor through within-fold screening. The varying feature sets across folds reflect genuine inter-fold variability rather than methodological error, and performance estimates remain unbiased because test subjects never contribute to selection decisions.

### 2.7 Microbiome feature representations

Two complementary microbiome feature strategies were evaluated, representing different approaches to dimensionality reduction and biological hypothesis testing:

#### 2.7.1 Option A: ANCOM-BC2 differentially abundant taxa

**Rationale:** Differential abundance analysis identifies taxa whose abundances systematically differ between preterm and term births, providing biologically interpretable features enriched for causal signals or biomarker potential. This focused approach offers strong dimensionality reduction while retaining taxa most likely associated with PTB pathophysiology.

**Method:** Analysis of Compositions of Microbiomes with Bias Correction version 2 (ANCOM-BC2) was applied to detect differentially abundant genera [16]. ANCOM-BC2 was performed independently within each outer cross-validation fold using only training samples. Taxa achieving nominal (rather than statistical) significance (p<0.10) within each fold’s training set were retained as features for model training in that fold. The liberal p-value threshold of 0.10 was chosen to ensure that microbial features were included in all folds, given that the global analysis showed relatively modest effect sizes and recognizing that overly stringent filtering in small training sets could result in folds with no selected microbiome features, precluding meaningful model evaluation. Hence, p-values in the present context are distribution descriptors rather than significance thresholds. Global descriptive power will be calculated later on via posterior cross-validation AUROC tests.

**Implementation Details:**

- **Global analysis (full cohort):** ANCOM-BC2 applied to all 43 subjects using absolute abundance data (read counts) from the decontaminated genus table (87 genera, prior to prevalence filtering) with adjustment for maternal age and pre-pregnancy BMI. Significance threshold: p<0.10 for identifying candidate taxa, acknowledging exploratory objectives and limited power. False discovery rate (FDR) correction using Benjamini-Hochberg method applied and reported.
- **Within-fold analysis (for machine learning):** To maintain leakage-aware framework, ANCOM-BC2 was executed *independently within each outer training fold* using only training subjects. Taxa achieving p<0.10 within that fold were selected as features for model training in that iteration. This approach ensures test subjects never influence feature selection and provides assessment of taxa stability across resampling.

#### 2.7.2 Option B: full filtered microbiome profile

**Rationale:** Univariate differential abundance testing may miss taxa involved in multivariate interactions, synergistic effects, or subtle compositional shifts detectable only through ensemble modeling. Including all prevalence-filtered genera maximizes information content and allows machine learning algorithms to discover complex patterns not apparent in univariate analysis.

**Implementation:** All 49 prevalence-filtered genera (present in ≥5% of samples) were included as CLR-transformed abundances plus Shannon diversity index (60 features total). This comprehensive approach tests whether high-dimensional microbiome representation improves discrimination despite increased risk of overfitting with n=43.

**Trade-off:** Higher feature dimensionality increases overfitting potential but enables detection of multivariate microbiome signatures and taxa co-occurrence patterns. Regularization in Elastic Net and bootstrap aggregation in Random Forest provide some protection against overfitting.

### 2.8 Machine learning algorithms

We selected two complementary supervised learning algorithms with established track records in small-sample microbiome studies and complementary properties for pattern detection:

#### 2.8.1 Random forest

Random Forest [17], an ensemble method combining predictions from hundreds of bootstrap-aggregated decision trees, was selected for its:

- Proven performance in the DREAM Challenge microbiome-PTB prediction task [8]
- Demonstrated reliability in microbiome studies with n<150 [18]
- Native handling of high-dimensional feature spaces through random feature subsampling at each tree split
- Robustness through ensemble averaging reducing overfitting
- Ability to capture non-linear relationships and complex interactions without explicit feature engineering

**Hyperparameter Selection:** Hyperparameters were fixed *a priori* rather than optimized through grid search, following established best practices for small-sample studies to reduce overfitting risk and enhance reproducibility:

- **Number of trees (ntree):** 500 trees. Standard choice providing stable estimates; performance typically plateaus beyond 500 trees [17]
- **Number of features per split (mtry):** 4 features. Approximates $\sqrt{\;p}$ for p≈14−70 features (depending on feature selection strategy), balancing tree diversity and accuracy following Breiman’s recommendation [17]
- **Minimum node size (min_n):** 10 observations. Conservative threshold (∼25% of training fold size) preventing overfitting to individual cases

These hyperparameters were selected based on: (1) theoretical recommendations from original Random Forest publications; (2) empirical performance across diverse datasets demonstrating near-optimal performance for default settings [19]; (3) computational feasibility and reduced energy consumption. Extensive hyperparameter tuning with n=43 would risk overfitting and provide marginal gains while substantially complicating the analysis.

**Implementation:** Random Forest models trained using the ranger package (v0.16.0) in R through the tidymodels framework [20].

#### 2.8.2 Elastic net logistic regression

Elastic Net [21] combines L1 (LASSO) and L2 (Ridge) regularization penalties, providing:

1. Linear interpretability through direct coefficient quantification (facilitating biological interpretation)
2. Built-in feature selection via L1 penalty (coefficients of uninformative features shrink to exactly zero)
3. Multicollinearity handling through L2 penalty (grouping correlated features)
4. Explicit regularization preventing overfitting

**Hyperparameter Selection:**

- **Mixing parameter (α):** 0.5 (equal weighting of L1 and L2 penalties). This balanced approach combines LASSO’s feature selection with Ridge’s stability for correlated predictors
- **Regularization parameter (λ):** 0.01 (moderate regularization strength), specified a priori as a conventional value providing meaningful coefficient shrinkage while retaining informative features—a deliberate choice given the demonstrated instability of cross-validation-selected λ in small samples [22, 23]. A sensitivity analysis confirmed stable performance at λ=0.001 and λ=0.01, while λ=0.1 produced excessive regularization leading to model collapse in several folds (Supplementary Table S6).

**Implementation:** Elastic Net models trained using the glmnet package (v4.1-8) through the parsnip interface [24].

**Justification for Fixed Hyperparameters:** We deliberately chose fixed hyperparameters over data-driven optimization for several reasons:

- **Overfitting prevention:** With only 43 subjects and ∼34 training subjects per fold, hyperparameter tuning on the same data used for evaluation would yield inflated performance estimates
- **Reproducibility:** Fixed parameters enable exact replication and eliminate researcher degrees of freedom
- **Comparability:** Consistent hyperparameters across feature selection strategies isolate the effect of feature engineering from algorithm tuning
- **Computational efficiency:** Nested cross-validation with hyperparameter grid search would require thousands of model fits, unjustified for exploratory n=43 study
- **Established precedent:** The DREAM Challenge and similar benchmarking studies use consensus hyperparameters rather than sample-specific optimization to ensure fair comparisons [8]

This conservative approach prioritizes honest performance estimation and methodological transparency over marginal performance gains from optimization.

**Algorithms Explicitly Avoided:** We did not evaluate more complex algorithms (XGBoost, gradient boosting machines, deep neural networks) because these methods typically require n≥200−500 for reliable performance and carry high overfitting risk with n=43 [25].

### 2.9 Nested cross-validation framework

To ensure unbiased estimates of internal cohort structure and prevent information leakage, we implemented a rigorous two-loop nested cross-validation design that strictly separates threshold optimization from performance evaluation [9]. This framework addresses the critical methodological flaw common in small-sample studies: using test data for multiple purposes (threshold selection *and* performance evaluation), which inflates estimates and yields non-generalizable results.

#### 2.9.1 Outer loop: performance evaluation

**Purpose:** Provide honest, unbiased estimates of model discrimination reflecting internal cohort structure.

**Implementation:** Five-fold stratified cross-validation at *subject level* (not sample level). Stratification maintained PTB prevalence (∼33%) in each fold. Critically, **all samples from a given subject were assigned to the same fold**, preventing information leakage from longitudinal repeated measures.

**Rationale for Subject-Level Splitting:** Multiple samples per subject are not independent—they share host genetics, environment, immune status, and time-invariant characteristics. Sample-level splitting (randomly assigning samples to folds) would allow model to “memorize” subject-specific patterns from training samples and artificially inflate performance on test samples from the same subject. Subject-level splitting simulates real clinical deployment: predicting outcomes for *new patients* rather than new samples from known patients.

**Cross-Validation Structure:**

- Total: 43 subjects (14 PTB, 29 term)
- Folds 1–4: 9 subjects each (3 PTB, 6 term; 33.3% PTB prevalence)
- Fold 5: 7 subjects (2 PTB, 5 term; 28.6% PTB prevalence)
- Training set per iteration: ∼34 subjects (79% of cohort)
- Test set per iteration: ∼9 subjects (21% of cohort)

**Rationale for 5-Fold CV:** With only 43 subjects, 5-fold CV provides reasonable balance between training set size (34 subjects), test set size (9 subjects with 2–3 PTB cases enabling meaningful sensitivity/specificity calculation), and number of independent evaluations (5 iterations). Alternative strategies were considered but rejected: 10-fold CV would yield test sets with ∼4 subjects and potentially zero PTB cases in some folds; leave-one-out CV (LOOCV) would have extremely high variance and inability to aggregate multi-sample predictions.

**Process for Each Outer Fold:**

1. Partition subjects into training (79%) and test (21%) sets
2. Apply all preprocessing to training data: CLR transformation, feature selection (ANCOM-BC2 or univariate screening for data-driven approach), missing value handling
3. Execute inner loop (threshold optimization) using training subjects only
4. Train final model on outer training set using inner-optimized threshold
5. Generate predictions for outer test subjects
6. For subjects with multiple samples: average predicted probabilities across samples to obtain subject-level prediction
7. Calculate performance metrics on subject-level predictions
8. Store fold-specific results

No information from outer test subjects influenced model training, feature selection, or threshold optimization at any stage.

#### 2.9.2 Inner loop: threshold optimization

**Purpose:** Determine optimal classification threshold for converting predicted probabilities to binary class assignments, without touching outer test data.

**Implementation:** Within each outer training fold (34 subjects):

1. Further stratified 70/30 split at subject level:
  - Inner training set: ∼24 subjects (70%)
  - Inner validation set: ∼10 subjects (30%)
2. Train model on inner training set (24 subjects)
3. Generate predicted probabilities for inner validation set (10 subjects)
4. Aggregate multi-sample predictions to subject level (average probabilities)
5. Compute ROC curve on validation predictions
6. Calculate Youden’s Index at all possible thresholds: J=Sensitivity+Specificity−1
7. Select threshold maximizing Youden’s Index (balances true positive and true negative rates)
8. **Freeze this threshold**—it becomes fixed for the outer test fold

**Apply Frozen Threshold to Outer Test Fold:** Use inner-optimized threshold (not re-optimized on test data!), then apply threshold to outer test predictions, to finally calculate the performance metrics (AUROC, sensitivity, specificity, etc.)

This rigorous workflow establishes methodological best practices for future studies as sample sizes increase and external validation cohorts become available. The nested structure, while computationally intensive, provides honest performance estimates free from optimistic bias that has plagued smaller studies.

#### 2.9.3 Handling of longitudinal samples in cross-validation

The heterogeneous number of samples per subject (range 1–6) required careful handling during cross-validation:

1. **Fold assignment:** All samples from a subject assigned to the same fold (either training or test, never split)
2. **Model training:** All samples from training subjects used for model fitting (treating repeated measures as independent observations during training)
3. **Prediction aggregation:** For subjects with multiple samples in test set, predicted probabilities averaged across samples to obtain single subject-level prediction
4. **Performance calculation:** Metrics computed at subject level (one prediction per subject), not sample level

This strategy balances utilizing all available data (multiple samples improve within-subject characterization) while preventing leakage (strict subject-level partitioning) and generating clinically relevant predictions (subject-level outcomes).

**Sensitivity Analysis for Non-Independent Sampling Bias:** Given the opportunistic longitudinal design, term subjects contributed more samples on average (3.0 samples/subject) than PTB subjects (1.7 samples/subject). To rigorously test whether this class-imbalanced sampling depth skewed feature identification toward patterns overrepresented by term subjects with multiple visits, we conducted a sensitivity analysis implementing inverse-frequency sample weighting. In this approach, all longitudinal samples were retained, but case weights were applied during model training. Each sample received a weight of 1/n (where n is the total number of samples contributed by that subject), ensuring that every subject contributed exactly a total weight of 1.0 to the algorithm’s learning phase. The rationale and results of this sensitivity analysis, which confirmed the robustness of our unweighted primary approach, are detailed in the Supplementary Material (Sensitivity Analysis 5).

### 2.10 Model evaluation metrics

Model performance was assessed using multiple complementary metrics, with primary emphasis on discrimination rather than calibration given the exploratory descriptive framing:

- **Area Under Receiver Operating Characteristic Curve (AUROC):** Primary metric quantifying discrimination ability across all classification thresholds. Values range 0.5 (no discrimination) to 1.0 (perfect separation). AUROC > 0.7 considered acceptable, >0.8 excellent in clinical contexts, though interpretation requires population-specific calibration.
- **Area Under Precision-Recall Curve (PRAUC):** Emphasizes performance in class-imbalanced settings by focusing on positive class (PTB) precision and recall. More informative than AUROC when negative class (term births) substantially outnumbers positive class.
- **Sensitivity (Recall, True Positive Rate):** Proportion of PTB cases correctly identified. Critical for screening applications where missing PTB (false negatives) carries high clinical cost.
- **Specificity (True Negative Rate):** Proportion of term births correctly identified. Important for minimizing false alarms (false positives) that could lead to unnecessary interventions.
- **Balanced Accuracy:** Average of sensitivity and specificity, providing overall performance metric robust to class imbalance.

Performance metrics were computed independently for each of 5 outer cross-validation folds, then aggregated as mean ± standard deviation across folds. Standard deviations reflect genuine model performance variability due to fold composition with limited sample size, not methodological error. We report distributions rather than point estimates to convey uncertainty appropriately.

**Critical Interpretive Note:** AUROC and other discrimination metrics in this study quantify *internal cohort structure*—the degree to which clinical and microbiome features systematically associate with PTB outcome within this specific Mexican cohort. These metrics do *not* estimate external predictive performance, which would require validation in large, independent cohorts. The moderate-to-good discrimination observed indicates non-random patterning but should not be conflated with clinical utility or generalizability.

**Permutation Testing for Statistical Significance:** To formally test whether the observed model discrimination significantly exceeded what could be expected by random chance given the small sample size and class imbalance, we performed rigorous permutation testing on our best-performing model configuration. We shuffled the PTB/term outcome labels 500 times and ran each permuted dataset through the complete nested cross-validation pipeline. Crucially, to prevent data leakage during this process, label shuffling was performed strictly at the *subject level*, ensuring that all longitudinal samples from a given participant always received the same permuted label, thereby preserving the dataset’s correlation structure. A null distribution of AUROC values was generated, and an empirical p-value was calculated as the proportion of permuted AUROCs that equaled or exceeded the AUROC of the true model (Supplementary Figure S1). The empirical p-value was calculated using the conservative estimator of Phipson and Smyth [26]. The complete R code for this computationally intensive permutation test, along with all nested cross-validation workflows, is publicly available in the project’s repository at https://github.com/martinruhle/Mexican-PretermBirth-analysis. Per-fold performance metrics for this model configuration are reported in Supplementary Table S2.

### 2.11 Statistical analysis and software

All analyses were conducted in R version 4.4.2. Differential abundance analysis used the ANCOMBC package (v2.0.2). Machine learning workflows implemented via tidymodels framework. Principal component analysis performed on CLR-transformed abundances using prcomp. Cohort characteristics compared between PTB and term groups using *t*-tests (continuous variables) or Fisher’s exact tests (categorical variables). Statistical significance defined as p<0.05 unless otherwise specified. Complete analysis code, including nested cross-validation workflows and ANCOM-BC2 implementation, is publicly available at https://github.com/martinruhle/Mexican-PretermBirth-analysis.

## 3 Results

### 3.1 Cohort characteristics and clinical variables

The analysis cohort comprised 43 pregnant Mexican women contributing 110 vaginal microbiome samples collected longitudinally throughout pregnancy. Fourteen participants (32.6%) experienced preterm birth (<37 weeks’ gestation), while 29 (67.4%) delivered at term (≥37 weeks). Table 1 summarizes demographic, anthropometric, and clinical characteristics stratified by pregnancy outcome.

**Table 1:** Cohort characteristics stratified by pregnancy outcome.

Characteristic	PTB (n=14)	Term (n=29)	p-value
*Demographics*			
Maternal age (years)	25.2±7.3	24.2±4.9	0.6
Advanced age (≥35 years), n (%)	2 (14.3)	1 (3.6)	0.3
Education level, n (%)			0.073
Primary or less	2 (14.3)	0 (0)	
Secondary	6 (42.9)	13 (46.4)	
Vocational/technical	5 (35.7)	15 (53.6)	
Associates or higher	1 (7.1)	0 (0)	
*Anthropometrics*			
Pre-pregnancy weight (kg)	62.4±13.4	58.6±9.8	0.3
Height (cm)	156.4±6.6	156.6±6.3	>0.9
Pre-pregnancy BMI (kg/m${}^{2}$)	25.6±5.6	23.9±3.5	0.3
BMI category, n (%)			0.012
Underweight (<18.5)	2 (14.3)	0 (0)	
Normal (18.5–24.9)	5 (35.7)	17 (60.7)	
Overweight (25.0–29.9)	3 (21.4)	10 (35.7)	
Obese (≥30.0)	4 (28.6)	1 (3.6)	
*Pregnancy Outcomes*			
GA at delivery (weeks)	33.7±4.1	39.3±1.1	<0.001
Range (weeks)	20.0–36.4	37.0–41.6	
PTB timing, n (%)			
Very early (<28 wk)	1 (7.1)	–	
Moderate (32–33 wk)	4 (28.6)	–	
Late (34–36 wk)	9 (64.3)	–	
Birth weight (g)	2,435.8±353.0	3,143.0±428.8	<0.001
Low birth weight (<2,500 g), n (%)	7 (58.3)	1 (3.6)	<0.001
*Obstetric Complications, n (%)*			
First trimester bleeding	1 (7.1)	8 (27.6)	0.2
Preeclampsia	1 (7.1)	0 (0)	0.3
PPROM	3 (21.4)	0 (0)	0.029
Term PROM	0 (0)	5 (17.2)	0.2
Oligohydramnios	2 (14.3)	1 (3.4)	0.2
IUGR	1 (7.1)	0 (0)	0.3
Gestational diabetes	0 (0)	0 (0)	–
Anemia (any visit)	2 (14.3)	7 (24.1)	0.7
Hemoglobin (g/dL)	11.8±0.9	11.5±0.7	0.5
*Sampling Structure*			
Number of visits			0.052
1 visit, n (%)	8 (57.1)	4 (13.8)	
2–3 visits, n (%)	5 (35.7)	17 (58.6)	
4+ visits, n (%)	1 (7.1)	8 (27.6)	
GA at first visit (weeks)	21.0±6.5	18.6±5.8	0.2
GA at last visit (weeks)	26.3±6.8	31.8±6.4	0.018

**Demographics and Anthropometrics:** Mean maternal age was 24.5±5.8 years overall, with no significant difference between PTB (25.2±7.3 years) and term (24.2±4.9 years) groups (p=0.6). Only 7.1% of participants were ≥35 years (advanced maternal age). Pre-pregnancy body mass index averaged $24.4\pm 4.3\;{\text{kg/m}}^{2}$ (PTB: 25.6±5.6; term: 23.9±3.5; p=0.3), though BMI category distribution differed between groups (p=0.012). Notably, underweight (BMI < 18.5) and obesity (BMI ≥ 30) were more prevalent in the PTB group (14.3% and 28.6%, respectively) compared to term group (0% and 3.6%), while normal-weight status was less common in PTB (35.7% vs. 60.7%).

**Pregnancy Outcomes:** Gestational age at delivery differed markedly between groups (PTB: 33.7±4.1 weeks, range 20.0–36.4; term: 39.3±1.1 weeks, range 37.0–41.6; p<0.001). Among 14 PTB cases: 1 very early preterm (<28 weeks, 7.1%), 4 moderate preterm (32–33 weeks, 28.6%), and 9 late preterm (34–36 weeks, 64.3%). The predominance of late preterm births reflects typical PTB distribution but limits inference regarding microbiome signatures specific to early deliveries.

Birth weight was significantly lower in PTB group (2,435.8±353.0 g) compared to term (3,143.0±428.8 g; p<0.001), with 58.3% of PTB infants classified as low birth weight (<2,500 g) vs. 3.6% of term infants (p<0.001).

**Obstetric Complications:** Preterm premature rupture of membranes (PPROM) occurred significantly more frequently in PTB group (21.4%) compared to term group (0%, p=0.029), consistent with established PTB risk factors. Other complications showed numerical differences without reaching statistical significance given limited sample size: preeclampsia (PTB: 7.1% vs. term: 0%, p=0.3), oligohydramnios (PTB: 14.3% vs. term: 3.4%, p=0.2), IUGR (PTB: 7.1% vs. term: 0%, p=0.3). No participants developed gestational diabetes. First trimester bleeding paradoxically showed higher prevalence in term group (27.6%) than PTB group (7.1%, p=0.2), though this difference was also not statistically significant.

**Hematological Parameters:** Mean hemoglobin concentration was similar between groups (PTB: 11.8±0.9 g/dL; term: 11.5±0.7 g/dL; p=0.5). Anemia (hemoglobin <11 g/dL at any visit) prevalence did not differ significantly (PTB: 14.3%, term: 24.1%, p=0.7).

**Sociodemographic Characteristics:** Educational attainment distribution showed no significant differences (p=0.073), though PTB group had higher representation at extremes (primary education or less: 14.3% vs. 0%; associates degree or higher: 7.1% vs. 0%). Marital status and employment patterns were similar between groups.

**Longitudinal Sampling Structure:** Participants provided mean 2.6±1.4 samples (median 2, range 1–6). Number of samples per participant differed between groups (p=0.052), with PTB pregnancies more likely to have single samples (57.1% with 1 visit) compared to term pregnancies (13.8% with 1 visit), reflecting earlier delivery interrupting longitudinal follow-up. Gestational age at first visit was similar between groups (PTB: 21.0±6.5 weeks; term: 18.6±5.8 weeks; p=0.2), but gestational age at last visit differed significantly (PTB: 26.3±6.8 weeks; term: 31.8±6.4 weeks; p=0.018) due to preterm delivery.

Overall, univariate comparisons revealed substantial overlap between PTB and term groups for most clinical variables, with no single feature providing strong discrimination. BMI category distribution and PPROM showed statistically significant differences, while several obstetric complications exhibited numerical trends warranting consideration in multivariate models despite lack of univariate significance.

### 3.2 Vaginal microbiome compositional patterns

Following quality filtering and prevalence thresholding (genera present in ≥5% of samples), 59 bacterial genera were retained from an initial set of 97 genera across 110 samples. After computational decontamination (removal of 10 putative contaminant genera), 49 genera were retained for machine learning analyses, while the full decontaminated set (87 genera) was used for global differential abundance testing. Vaginal communities exhibited compositional patterns typical of pregnancy microbiomes, with high inter-individual variability.

**Dominant Taxa:** *Lactobacillus* dominated most samples, with median relative abundance 82.2% across all samples (interquartile range 52.8–92.3%). However, 26 samples (23.6%) exhibited reduced *Lactobacillus* dominance (<50% relative abundance), a pattern previously associated with bacterial vaginosis, vaginal dysbiosis, and adverse pregnancy outcomes. Other abundant genera included *Gardnerella* (median 5.5%, IQR 2.3–30.2%, present in 100% of samples), *Gordonia* (median 0.4%, detected in 61.8%), and *Peptostreptococcus* (median 0% reflecting zero-inflated distribution, detected in 21.8% of samples overall but 33.3% of PTB samples vs. 16.3% of term samples).

**Alpha Diversity:** Shannon diversity index, calculated from untransformed relative abundances (prior to CLR transformation), ranged from 0.05 to 1.82 (mean ± SD: 0.76±0.51) across all samples. Counter to some prior studies, mean Shannon diversity did not differ significantly between PTB (0.81±0.60) and term (0.75±0.48) samples (*t*-test p=0.57), suggesting overall community richness and evenness were comparable between outcome groups. The association between diversity and PTB may be outcome-timing-dependent or population-specific.

**Figure 2:**
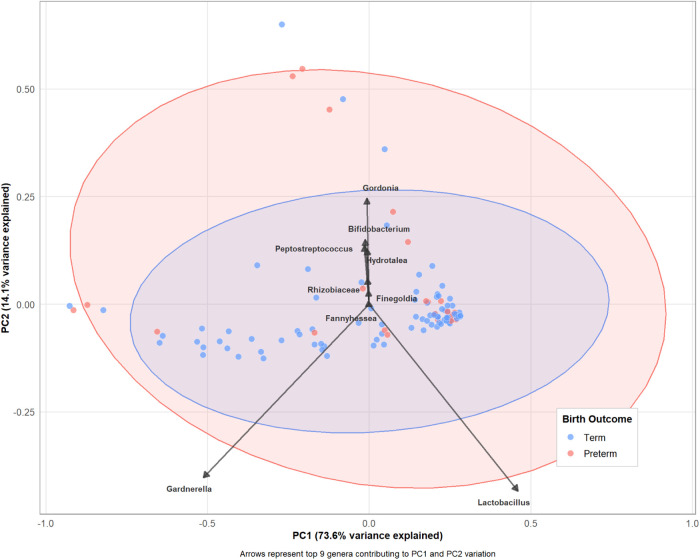
Principal component analysis of vaginal microbiome composition. PCA biplot of CLR-transformed genus-level abundance data showing sample-level compositional patterns (colored points) and major taxonomic drivers of variation (arrows). Each point represents one vaginal microbiome sample colored by birth outcome (blue = term, red = preterm). Arrows indicate the top 10 contributing genera to PC1 and PC2 variation, with arrow length and direction reflecting magnitude and sign of each genus’s contribution. Shaded ellipses represent 95% confidence regions for each outcome group, demonstrating complete overlap with greater dispersion in PTB samples. PCA performed on centered log-ratio (CLR)-transformed abundances using pseudocount 0.65 to handle compositional zeros.

**Compositional Structure:** Principal component analysis (PCA) of CLR-transformed abundance data revealed broad dispersion of samples across the first two components, which together explained 87.7% of total microbiome compositional variance (PC1: 73.6%, PC2: 14.1%; Figure 2). Samples clustered primarily by dominant taxon (strong *Lactobacillus* dominance vs. dysbiotic communities), with a complete overlap between PTB and term samples. Visual inspection confirmed no clear separation of outcome groups based solely on microbiome composition, consistent with moderate rather than strong discrimination in supervised analyses. The top contributing genera to PC1 were *Lactobacillus* (negative loading, dominant communities) and *Gardnerella*, *Prevotella*, *Atopobium* (positive loadings, dysbiotic communities), reflecting the primary axis of variation from eubiotic to dysbiotic states.

### 3.3 Global differential abundance analysis

**Figure 3:**
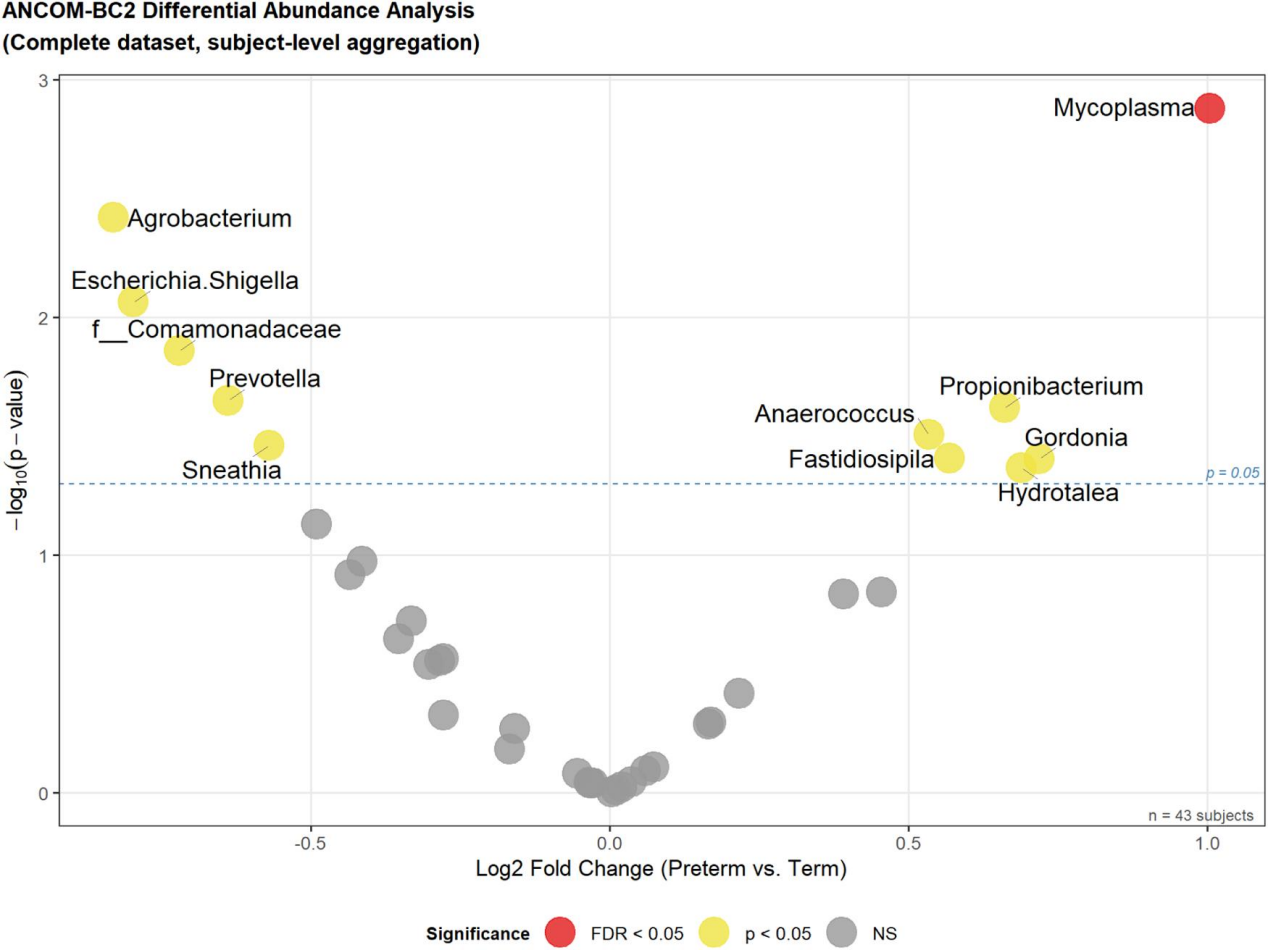
Global differential abundance analysis by ANCOM-BC2. Volcano plot showing log2 fold change (preterm vs. term) against $-\log_{10}$ (p-value) for 87 genera in the decontaminated dataset (43 subjects). ANCOM-BC2 was adjusted for maternal age and pre-pregnancy BMI. Horizontal dashed lines indicate nominal significance thresholds (p=0.05, p=0.10) and the empirical FDR = 0.05 boundary. Point colors indicate significance level.

ANCOM-BC2 analysis conducted on the decontaminated dataset (43 participants, 87 genera) adjusted for maternal age and pre-pregnancy BMI identified one genus achieving FDR-corrected significance at q < 0.05 (Figure 3): *Mycoplasma* (LFC =+1.004, p=0.001, q=0.049), enriched in PTB samples. *Agrobacterium* approached FDR significance (LFC =−0.830, q=0.070, PTB-depleted).

Ten additional genera reached nominal significance (p<0.05) without surviving FDR correction, including five PTB-depleted taxa—*Escherichia-Shigella* (LFC =−0.797, p=0.009), *Comamonadaceae* (LFC =−0.720, p=0.014), *Prevotella* (LFC =−0.638, p=0.022), *Sneathia* (LFC =−0.570, p=0.035)—and five PTB-enriched taxa—*Propionibacterium* (LFC =+0.661, p=0.024), *Anaerococcus* (LFC =+0.534, p=0.031), *Fastidiosipila* (LFC =+0.569, p=0.039), *Gordonia* (LFC =+0.719, p=0.039), and *Hydrotalea* (LFC =+0.689, p=0.043). *Lactobacillaceae* showed a trend toward PTB depletion (LFC =−0.490, p=0.074).

The directionality of nominally significant associations is biologically coherent: PTB enrichment of *Mycoplasma*—a genus established in reproductive tract infection and chorioamnionitis [27, 28]—alongside nominal depletion of *Prevotella*, *Sneathia*, and *Lactobacillaceae*, suggests a shift in vaginal community structure associated with PTB. The depletion of *Escherichia-Shigella* in PTB warrants cautious interpretation given that this genus encompasses both commensal and pathogenic lineages, and 16S V3-V4 resolution cannot distinguish among them.

### 3.4 Machine learning model performance and internal discrimination

Twelve model configurations were systematically evaluated, representing combinations of 2 algorithms (Random Forest, Elastic Net) × 3 clinical feature selection approaches (DREAM-style, literature-based, data-driven) × 2 microbiome representations (ANCOM-selected taxa, full filtered profile). Performance metrics for the top 5 ranked models are presented in Table 2, with complete results for all 12 model configurations available in Supplementary Table S1. Performance varied substantially across configurations, with Random Forest consistently outperforming Elastic Net (mean AUROC 0.737 vs. 0.668 across all RF and Elastic Net models, respectively).

**Table 2:** Performance of top 5 ranked PTB prediction models.

Algorithm	Features	Microbiome	AUROC	PRAUC	Sensitivity	Specificity
RF	Data-driven	Full	0.813±0.110	0.592±0.209	0.600±0.365	0.613±0.289
RF	Data-driven	ANCOM taxa	0.791±0.170	0.600±0.208	0.533±0.380	0.553±0.299
EN	Literature	ANCOM taxa	0.789±0.190	0.803±0.210	0.200±0.298	0.493±0.325
EN	Data-driven	ANCOM taxa	0.784±0.067	0.566±0.165	0.600±0.365	0.653±0.238
EN	Literature	Full	0.760±0.117	0.814±0.171	0.200±0.298	0.627±0.293

Values represent mean ± SD across 5-fold nested cross-validation. Features: DREAM = gestational age + maternal age; Literature = 10 evidence-based PTB risk factors; Data-driven = top 10 variables by univariate association (selected per fold). Microbiome: ANCOM taxa = differentially abundant genera (p<0.10); Full = all 49 filtered genera. Classification thresholds optimized on inner validation sets using Youden’s Index. RF = Random Forest; EN = Elastic Net. Complete results for all 12 model configurations in Supplementary Table S1.

Random Forest models generally outperformed Elastic Net across feature selection strategies, consistent with ensemble methods’ robustness to correlated predictors and nonlinear relationships. Among Random Forest implementations, data-driven feature selection yielded higher AUROC than either minimal DREAM-style adjustment (AUROC 0.751±0.087) or literature-based features (AUROC 0.709±0.135 with full microbiome), suggesting that population-specific empirical selection captures locally relevant risk factors.

ANCOM-BC2 filtered microbiome (selecting only differentially abundant taxa) provided comparable or superior performance to full microbiome profiles in several configurations, demonstrating that hypothesis-driven feature selection can improve signal-to-noise ratio despite reducing dimensionality.

**Figure 4:**
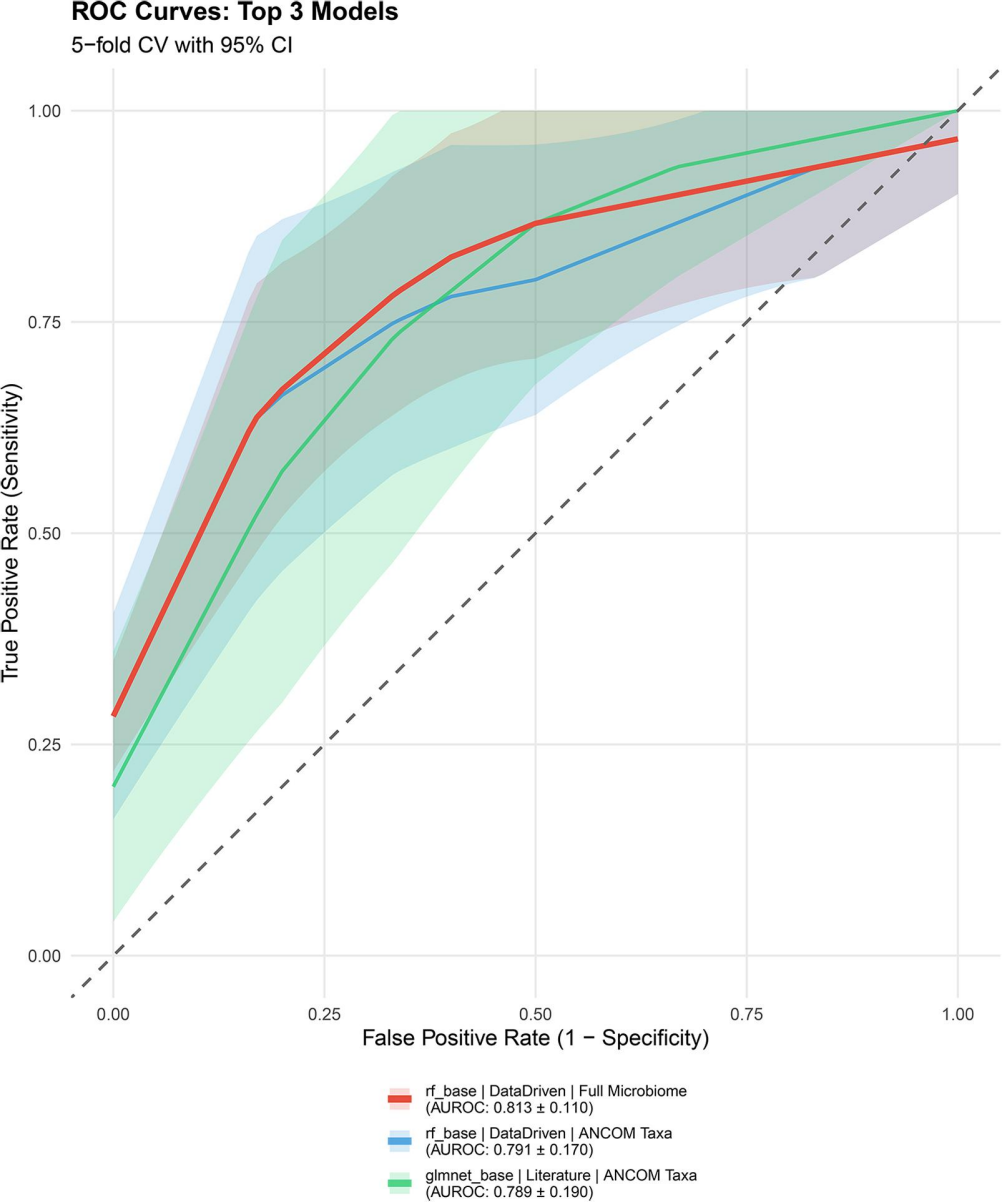
ROC curves for top-performing models. Receiver Operating Characteristic (ROC) curves showing true positive rate (sensitivity) vs. false positive rate (1-specificity) across classification thresholds. Curves represent mean performance across 5 cross-validation folds for the three best-performing model configurations: Random Forest with data-driven feature selection and full microbiome (AUROC 0.813), Random Forest with data-driven features and ANCOM taxa (AUROC 0.791), and Elastic Net with literature-based features and ANCOM taxa (AUROC 0.789). Shaded regions indicate fold-to-fold variability (mean ± SD). The diagonal dashed line represents random chance (AUROC = 0.5). All models substantially exceed chance performance, though wide confidence bands reflect genuine uncertainty derived from small sample size.

ROC curves for the top 3 models (Figure 4) illustrate discrimination performance across the full threshold range, with confidence regions highlighting fold-to-fold variability.

### 3.5 Feature importance and selection stability

**Figure 5:**
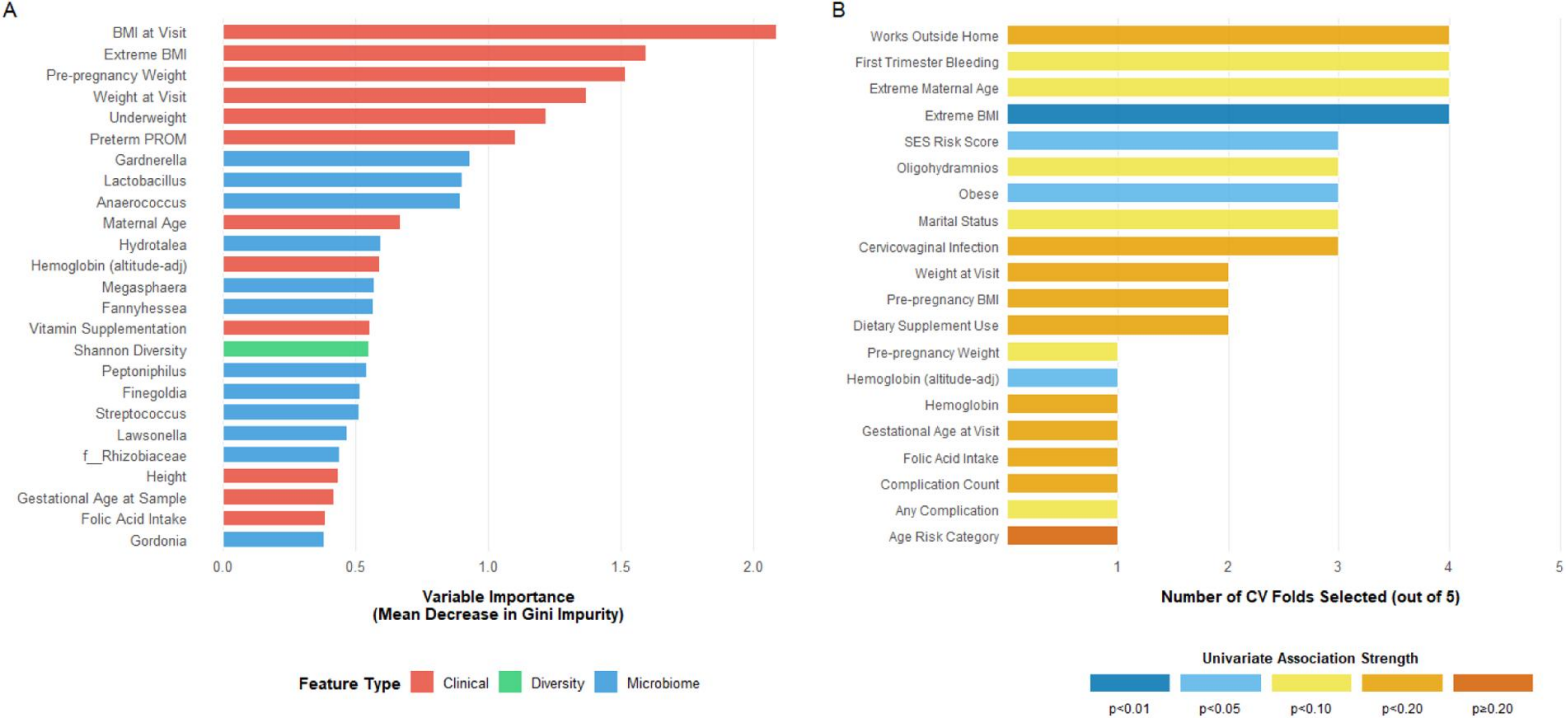
Feature importance and selection stability in best-performing random forest model. **(A)** Variable importance for top 25 predictive features based on mean decrease in Gini impurity, aggregated across 5 cross-validation folds for Random Forest with data-driven feature selection and full microbiome representation (best-performing model configuration). Features colored by type: clinical variables (red), microbial genera (blue), diversity metrics (green). **(B)** Selection stability of clinical features across 5 cross-validation folds using data-driven univariate screening strategy (top 10 features selected per fold based on strongest univariate PTB associations). Bar height indicates selection frequency (0–5 folds, proportion labeled); bar color represents univariate association strength in folds where selected. Note: Microbiome features not shown in panel B as full microbiome approach (all 49 genera) was used in all folds without feature selection.

Feature importance analysis for the best-performing Random Forest model (data-driven features, full microbiome) revealed that anthropometric variables dominated predictive signal (Figure 5). Pre-pregnancy BMI, BMI at sample collection visit, and pre-pregnancy weight ranked among the top 5 most important features, consistent with epidemiological evidence linking maternal body composition extremes to preterm birth risk.

Among microbiome features, *Peptostreptococcus*, *Mycoplasma*, and *Lactobacillus* exhibited highest importance scores, indicating consistent discriminative value across bootstrap resampling iterations. Gestational age at sampling, maternal age, and PPROM also contributed substantially, aligning with established PTB risk factors.

Feature selection stability analysis (Figure 5, panel B) demonstrated that no clinical features were selected in all 5 cross-validation folds, reflecting inter-fold variability expected with modest sample size. BMI-related variables (BMI category, extreme BMI indicator, pre-pregnancy BMI) and PPROM showed highest selection frequency (4/5 folds), representing the most robust empirical predictors. Features selected in fewer folds either have weaker univariate associations or are sensitive to fold composition.

### 3.6 Within-fold differential abundance and feature stability

**Figure 6:**
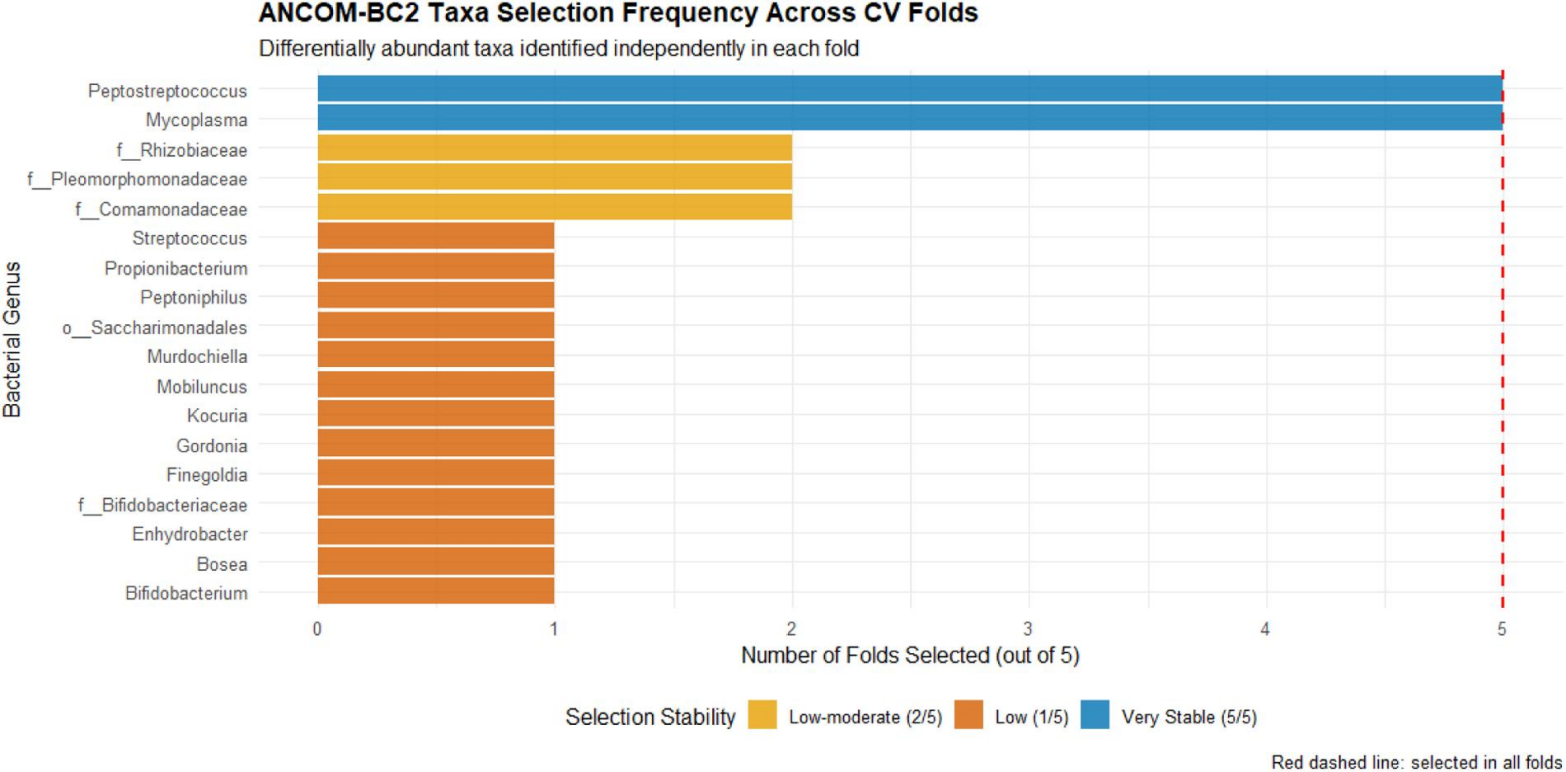
Differential abundance detection frequency across cross-validation folds. Bar plot showing number of cross-validation folds (out of 5 total) in which each genus was identified as differentially abundant between PTB and term samples by ANCOM-BC2 analysis (nominal p<0.10), conducted independently within each outer training fold to prevent data leakage. Only genera detected in ≥2 folds shown.

To assess the robustness of differential abundance signals beyond single global analyses, we examined detection frequency of taxa across the 5 independent ANCOM-BC2 analyses conducted within outer cross-validation folds (Figure 6) (Supplementary Table S3).

*Mycoplasma* was detected as differentially abundant (p<0.10, PTB-enriched) in 5/5 folds (100% stability), consistent with its FDR-corrected significance in the global analysis and representing the most robust microbiome signal in the cohort. *Peptostreptococcus* was also detected in 5/5 folds (100% stability) with consistent PTB enrichment, despite not reaching nominal significance in the global adjusted analysis (p=0.058). This discrepancy likely reflects that within-fold analyses, conducted on ∼34-subject training sets, are not adjusted for identical covariates as the global model and operate on different subsets where the *Peptostreptococcus* signal may be relatively stronger. The 100% within-fold detection provides complementary evidence of signal consistency that single-cohort global analyses—sensitive to specific covariate adjustment and sample composition—may not capture.

Convergent evidence from multiple independent analytical approaches—global differential abundance (FDR-corrected for *Mycoplasma*; within-fold replication for *Peptostreptococcus*), within-fold detection stability (100% for both taxa), and feature importance rankings (top-ranked microbiome features in best model)—supports genuine PTB associations for *Mycoplasma* and *Peptostreptococcus* warranting biological interpretation and validation in independent cohorts.

## 4 Discussion

### 4.1 Descriptive machine learning as a tool for cohort structure characterization

This study applied leakage-aware machine learning not as a predictive tool for clinical deployment, but as a *descriptive analytical framework* to characterize clinical and vaginal microbiome patterns associated with preterm birth in a relatively small Mexican pregnancy cohort. This explicit reframing is critical: the moderate-to-good internal discrimination observed (AUROC 0.813±0.110 for best model) indicates the presence of non-random structure linking clinical variables and vaginal microbiome composition to pregnancy outcome *within this specific cohort*. However, wide dispersion of performance estimates across cross-validation folds (SD 0.110, range 0.703–0.923) highlights instability inherent to limited sample size and reinforces the necessity of interpreting discrimination metrics as descriptors of internal cohort organization rather than estimates of external predictive performance.

This distinction is particularly important in the context of microbiome-based machine learning studies, where optimistic performance estimates frequently arise from subtle forms of information leakage or cohort-specific confounding [10]. Common sources include: (1) threshold optimization on test data, (2) feature selection using all subjects before splitting train/test, (3) sample-level rather than subject-level cross-validation with longitudinal data, and (4) insufficient hold-out of test subjects from all model development steps. By enforcing subject-level partitioning, nested cross-validation, and within-fold feature selection, the present analysis prioritizes methodological rigor over maximal apparent performance, accepting increased variance in metrics as the cost of honest estimation.

The observed AUROC of 0.813, while seemingly promising, should not be interpreted yet as evidence of clinical utility or readiness for deployment. This value quantifies *how well clinical and microbiome features organize participants into outcome-concordant groups within a 43-subject Mexican cohort*, not how accurately a model trained on these 43 subjects would perform on new, unseen Mexican pregnancies. External validation in substantially larger, independent cohorts (n ≥ 200–500) is an absolute prerequisite before any claims of predictive validity can be entertained. Until such validation occurs, our findings serve primarily as hypothesis-generating signals warranting replication rather than actionable clinical tools.

### 4.2 Feature recurrence and biological interpretation

Across resampling iterations, anthropometric variables—particularly pre-pregnancy BMI, pre-pregnancy weight, and BMI at visit—were consistently ranked among the most informative features, alongside gestational age at sampling. This recurrent prioritization suggests clinical covariates remain strong contributors to PTB-associated structure even when high-dimensional microbiome features are explicitly modeled. The prominence of BMI is consistent with epidemiological literature demonstrating U-shaped BMI-PTB relationships: both underweight (BMI < 18.5) and obese (BMI ≥ 30) women face elevated PTB risk compared to normal-weight women [29]. In our cohort, BMI category distribution differed significantly between outcomes (p=0.012), with PTB cases overrepresented at BMI extremes (14.3% underweight, 28.6% obese vs. 0% and 3.6% in term group).

Among microbiome features, *Mycoplasma* and *Peptostreptococcus* emerged as consistently important across feature importance analyses and within-fold differential abundance testing, with detection stability of 100% across cross-validation folds (Supplementary Table S3). Critically, while *Mycoplasma* achieved FDR-corrected significance in the global analysis (q=0.049), *Peptostreptococcus* did not reach nominal significance globally (p=0.058) yet was detected in all 5 within-fold ANCOM-BC2 analyses and ranked among the top microbiome features by importance. This pattern—where a taxon fails global testing but shows consistent within-fold signal—illustrates a known property of resampling-based approaches: subsample analyses can recover associations that are attenuated in the full dataset by specific covariate adjustment or by the influence of individual subjects with high leverage. The convergence of within-fold stability and feature importance constitutes the strongest hypothesis-generating signal in our dataset for both taxa. However, we emphasize that cross-validation stability within a single 43-subject cohort does not substitute for external validation or a powered test of association. The following mechanistic discussion contextualizes these genera within established reproductive biology but should be interpreted as identifying plausible biological hypotheses for future confirmatory studies, not as evidence of confirmed pathogenic roles in this population.

#### 4.2.1 Peptostreptococcus and genital tract inflammation

*Peptostreptococcus* is a strictly anaerobic Gram-positive coccus predominantly found in human fecal microbiota and oral cavities, but also isolated from vaginal samples, particularly in dysbiotic states. Multiple species exist (*P. anaerobius*, *P. stomatis*), though genus-level 16S resolution precludes species identification here.

*Peptostreptococcus* has been associated with bacterial vaginosis, a well-established PTB risk factor characterized by *Lactobacillus* depletion and anaerobic overgrowth [30]; with ascending intrauterine infection leading to chorioamnionitis and fetal inflammatory response [4]; and with pro-inflammatory metabolite production (short-chain fatty acids, ammonia) that can activate Toll-like receptors on cervical and decidual cells, inducing cytokine cascades (IL-6, IL-8, TNF-α) [31]. Some strains also produce proteases contributing to extracellular matrix degradation and cervical remodeling.

*Peptostreptococcus* has been identified in placental tissues associated with severe chorioamnionitis and adverse birth outcomes in a Malawian cohort [32], corroborating its potential pathogenic relevance across genetically and geographically diverse populations. Although *Peptostreptococcus* did not reach nominal significance in the global adjusted analysis (LFC =+0.455, p=0.058), its 100% detection rate across cross-validation folds (5/5 folds) with consistent PTB enrichment, combined with its ranking among the top microbiome features by variable importance, provides convergent evidence of a genuine albeit modest association. The attenuation in the global analysis likely reflects the relatively small effect size after adjustment for maternal age and BMI, which may partially mediate the microbiome–PTB relationship.

Notwithstanding these prior reports, the association observed in our cohort should be interpreted with caution given the limited sample size, absence of FDR-corrected significance in the global analysis, and genus-level taxonomic resolution that precludes species-level attribution.

#### 4.2.2 Mycoplasma and intrauterine infection

*Mycoplasma* comprises cell wall-deficient bacteria including species highly relevant to reproductive health: *Mycoplasma hominis*, *Mycoplasma genitalium*, and *Ureaplasma* species (sometimes classified separately but closely related).

These organisms are implicated in chorioamnionitis—being among the most frequently isolated bacteria from amniotic fluid in intrauterine infection [27]—and in preterm premature rupture of membranes through production of phospholipases and proteases that degrade fetal membrane collagen [28]. Mycoplasma lipoproteins trigger TLR2/TLR6 signaling, inducing prostaglandin synthesis and matrix metalloproteinase upregulation that can initiate preterm labor [4]. Transplacental passage or amniotic fluid colonization may also provoke fetal inflammatory response syndrome with adverse neonatal sequelae.

In our cohort, *Mycoplasma* was the sole genus achieving FDR-corrected significance in the global analysis (LFC =+1.004, q=0.049), representing approximately 2-fold enrichment in PTB samples. It was additionally detected in 100% of cross-validation folds (5/5), showing consistent PTB enrichment across independent data partitions. The detection of *Mycoplasma* in 33.3% of PTB samples vs. 16.3% of term samples (prevalence-based comparison) further supports differential colonization. Genus-level taxonomy precludes species identification—future studies employing shotgun metagenomic sequencing or species-specific qPCR should determine whether *M. hominis*, *M. genitalium*, or *Ureaplasma* drive observed associations, as these species exhibit distinct pathogenic mechanisms and antibiotic susceptibility profiles.

Despite being the only taxon surviving FDR correction, the *Mycoplasma* association should still be interpreted as hypothesis-generating given the constraints of sample size and the borderline q-value (0.049). Its consistency across both global and within-fold analyses, combined with strong biological plausibility, makes it the highest-priority target for confirmatory investigation in adequately powered cohorts.

#### 4.2.3 Mechanistic hypotheses and causal considerations

The recurrent detection of *Peptostreptococcus* and *Mycoplasma* provides biological plausibility but does *not* establish causality. Several alternative explanations warrant consideration:

- **Causal pathogens:** These bacteria directly cause or contribute to PTB through infection/inflammation pathways described above (ascending infection model).
- **Opportunistic colonizers:** Underlying maternal factors (e.g., immune dysfunction, cervical insufficiency, hormonal imbalances) create permissive environment for dysbiotic bacteria; microbiome changes are consequences rather than causes of PTB risk.
- **Markers of broader dysbiosis:** *Peptostreptococcus* and *Mycoplasma* may serve as indicators of overall vaginal ecosystem perturbation (loss of *Lactobacillus* dominance, increased pH, reduced lactic acid) rather than specific etiologic agents.
- **Reverse causation:** Impending PTB (triggered by non-microbial factors like placental dysfunction or cervical insufficiency) may alter vaginal environment (increased pH, mucus production, inflammatory exudate), secondarily promoting growth of anaerobic bacteria.
- **Confounding by unmeasured variables:** Socioeconomic factors, sexual behaviors, hygiene practices, antibiotic use, or dietary patterns could independently influence both microbiome composition and PTB risk, creating spurious associations.

Distinguishing among these scenarios requires: (1) prospective longitudinal studies with early pregnancy sampling (before clinical PTB symptoms) to establish temporal precedence; (2) experimental validation in animal models or ex vivo systems demonstrating causal mechanisms; (3) intervention trials testing whether microbiome-targeted therapies (e.g., probiotics, antibiotics, vaginal pH modulation) reduce PTB incidence; and (4) multi-omics integration (metatranscriptomics, metabolomics, host immune profiling) to map microbial functions and host responses.

### 4.3 Longitudinal sampling heterogeneity and temporal dynamics

The heterogeneous longitudinal sampling scheme—with participants contributing 1–6 samples collected opportunistically during routine prenatal care rather than at standardized gestational timepoints—reflects real-world clinical follow-up patterns but introduces analytical complexity and interpretive limitations.

**Methodological Handling:** Our subject-level cross-validation strategy ensures longitudinal information does not induce data leakage: all samples from a subject assigned to the same fold (training or test, never split), and predictions aggregated to subject level for performance evaluation. This design prevents the model from learning subject-specific patterns from earlier samples to predict later outcomes artificially. Within training folds, multiple samples per subject contribute to model fitting, effectively increasing the data available for learning microbiome patterns while maintaining subject-level independence in test sets.

**Temporal Inference Limitations:** The variable sampling timing and unequal numbers of samples per participant fundamentally preclude any inference about the temporal trajectory of the vaginal microbiome or its causal relationship with PTB in the present dataset. We cannot determine whether early pregnancy microbiome (first trimester) differs in predictive capacity from late pregnancy samples, nor can we assess microbiome stability vs. dynamic shifts within individuals. Some participants contributed samples spanning 20+ weeks of gestation, while others provided single timepoint snapshots, creating heterogeneity in temporal coverage.

**Potential Confounding:** Gestational age at sampling differed between outcome groups (PTB last visit: 26.3 weeks; term last visit: 31.8 weeks; p=0.018) because preterm delivery interrupts longitudinal follow-up. This timing difference could introduce confounding if microbiome composition naturally changes with gestational age independent of outcome, and PTB-associated samples are systematically earlier in pregnancy. We partially addressed this by including gestational age at sampling as a covariate in all models, though residual confounding may persist. A sensitivity analysis restricted to samples collected before 28 weeks of gestation confirmed that discriminative performance was preserved (best AUROC 0.840±0.146; Supplementary Table S5), indicating that the identified signals are not solely attributable to late-pregnancy sampling artifacts.

**Future Study Design:** Prospective studies designed specifically to capture microbiome dynamics should prioritize:

- **Standardized sampling timepoints:** One sample per trimester (weeks 10–12, 20–22, 30–32) enabling trajectory analysis and identification of temporal windows with maximal predictive signal
- **Increased sampling frequency in high-risk women:** Biweekly or monthly samples for women with prior PTB, short cervix, or other risk factors to capture microbiome shifts preceding preterm labor
- **Longitudinal statistical methods:** Growth mixture modeling, functional data analysis, or recurrent neural networks to model within-subject trajectories rather than treating repeated measures as independent observations
- **Integration with cervical length ultrasound:** Co-temporal microbiome and cervical length measurements to assess whether microbial dysbiosis precedes, accompanies, or follows cervical shortening

Within the constraints of opportunistic sampling, the present analysis treats longitudinal samples as correlated observations within subjects, aggregating information to characterize average microbiome state rather than temporal evolution. This limitation tempers conclusions about mechanistic timing and temporal precedence of microbiome-PTB associations.

### 4.4 Methodological contributions

This study exemplifies several methodological principles essential for credible small-sample machine learning research:

**Subject-level cross-validation:** By partitioning participants rather than samples, we prevented information leakage from longitudinal repeated measures—a common but often-overlooked source of optimistic bias in pregnancy microbiome studies.

**Nested cross-validation with fold-specific preprocessing:** Feature selection, differential abundance analysis, and CLR transformation were performed independently within each training fold, ensuring that no test set information influenced model training. This approach yields conservative performance estimates but prevents the inflated metrics that plague improperly validated studies.

**Multiple complementary feature selection strategies:** Rather than committing to a single approach, we systematically evaluated theory-driven (literature-based), data-driven (empirical screening), and benchmark-driven (DREAM-style) strategies, demonstrating that different approaches prioritize different aspects of the data and providing robustness checks on findings.

**Convergent evidence synthesis:** Findings were considered robust only when supported by multiple independent analyses (global differential abundance, within-fold stability, feature importance), reducing false discovery risk.

**Transparent limitations acknowledgment:** We explicitly frame our results as descriptive characterizations of internal cohort structure rather than validated predictive models ready for clinical deployment, deferring claims of clinical utility until external validation is achieved.

### 4.5 Population specificity and health equity implications

To our knowledge, this represents the first machine learning-based study of vaginal microbiome patterns in Mexican pregnant women. Mexican and broader Latin American populations exhibit distinct genetic backgrounds, dietary patterns, socioeconomic contexts, healthcare access, and microbial exposures compared to the predominantly European-ancestry and North American cohorts that dominate existing literature. Vaginal microbiome composition is known to vary substantially by ethnicity [7], with Hispanic/Latino women more likely to exhibit *Lactobacillus crispatus*-dominated Community State Type I or intermediate/dysbiotic profiles compared to non-Hispanic white women.

The underrepresentation of Latin American cohorts in microbiome-PTB research constitutes both a scientific limitation (hindering generalizability of findings) and a health equity concern (perpetuating disparities in precision medicine access). Preterm birth rates, socioeconomic determinants, and healthcare infrastructure differ markedly between high-income and low-to-middle-income countries, necessitating population-specific validation of any proposed biomarker or prediction tool.

This study addresses this gap by providing foundational data on vaginal microbiome composition in Mexican pregnancies, establishing stable feature associations warranting replication, and delineating a principled pathway toward future externally validated PTB prediction models. Key priorities for translation include: (1) Multi-center validation cohorts with standardized longitudinal sampling protocols, (2) Integration of established clinical predictors (cervical length, obstetric history) alongside microbiome features, (3) Species-level taxonomic resolution to distinguish pathogenic from commensal strains, (4) Mechanistic multi-omics studies linking microbiome composition to immune response and metabolic dysregulation, and (5) Ultimately, intervention trials testing whether microbiome-targeted therapies reduce PTB incidence.

Cost-effectiveness analyses comparing microbiome-based screening to existing clinical approaches will also be essential for assessing feasibility of implementation in resource-constrained public health systems.

### 4.6 Limitations and future directions

Several limitations temper the interpretation and generalizability of our findings:

**Limited sample size and unfavorable predictor-to-sample ratio:** With 43 participants and 14 PTB cases, statistical power for detecting small-to-moderate effect sizes is constrained. More critically, the best-performing Random Forest model operated on approximately 60–70 predictors (clinical variables plus full microbiome) with only ∼34 training subjects per fold, yielding a predictor-to-sample ratio well below the minimum commonly recommended for stable model estimation [23]. Despite nested cross-validation preventing direct information leakage, this unfavorable ratio means the observed AUROC of 0.813 may still represent a partially optimistic estimate of the true discriminative structure, as the algorithm may capture noise patterns that happen to correlate with outcome within the limited cohort. Wide confidence intervals on performance metrics (SD 0.110–0.250) and the instability of clinical feature selection across cross-validation folds—where no single clinical variable was selected in all five folds (Figure 5B)—provide direct evidence of this limitation. The feature instability suggests that the discriminative signal, while exceeding chance (permutation p=0.033), is weak relative to sampling variability and highly dependent on fold composition. These observations reinforce that findings should be considered hypothesis-generating rather than confirmatory, and that substantially larger cohorts (n≥150−200) are needed for stable effect size estimation and reliable external validation. A learning curve analysis would be informative but is not reported here given that it would be prohibitively noisy at the current sample size.

**Additionally, one participant delivered at exactly 20.0 weeks’ gestation**—a value that meets the WHO threshold for preterm birth (≥20 weeks) but represents a clear outlier relative to the PTB group mean (33.7±4.1 weeks). In a cohort of only 14 PTB cases, a single extreme observation can exert disproportionate influence on model boundaries and feature importance estimates. To quantify this influence, we performed a sensitivity analysis excluding this participant (revised cohort: n=42, 13 PTB cases). Discrimination was preserved and improved in several configurations (Supplementary Table S4), confirming that the identified clinical-microbiome patterns are not artifacts of this single data point.

**Taxonomic resolution:** Genus-level 16S rRNA profiling cannot distinguish between closely related species with divergent pathogenic potential (e.g., *Lactobacillus crispatus* vs. *L. iners*, *Mycoplasma hominis* vs. *Ureaplasma urealyticum*). Shotgun metagenomic sequencing or species-specific qPCR would provide higher resolution and enable functional pathway analysis.

**Opportunistic sampling:** Heterogeneous timing of sample collection across participants introduces variability and precludes analysis of within-participant longitudinal dynamics (e.g., microbiome stability vs. transition patterns). Future studies should implement protocol-mandated fixed-timepoint sampling (e.g., 12, 20, 28 weeks gestation) to enable trajectory modeling.

**Single-center recruitment from public health facilities:** Participants were recruited from two public facilities serving predominantly low-to-middle socioeconomic populations in Mexico City. Findings may not generalize to private healthcare settings, rural populations, or other regions of Mexico with distinct sociodemographic characteristics.

**Case-control design:** Nested case-control sampling introduces survival bias (PTB cases must survive to sample collection) and precludes estimation of absolute risk or population-level predictive values. Prospective cohort designs with unselected recruitment enable more robust inference.

Future work should prioritize: (1) Larger multi-center cohorts with diverse socioeconomic representation, (2) Species-level microbiome profiling and functional metagenomics, (3) Integration of complementary biomarkers (host transcriptomics, cervical length, immune mediators), (4) Longitudinal modeling of microbiome dynamics, and (5) External validation in independent Mexican and Latin American cohorts before considering clinical translation.

### 4.7 Conclusions

By prioritizing methodological rigor and descriptive interpretation over premature predictive claims, this analysis provides a transparent and reproducible reference for exploratory microbiome research in pregnancy complications. To our knowledge, this represents the first application of machine learning methods to vaginal microbiome data from Mexican pregnant women, addressing a critical health equity gap. Within the constraints imposed by modest sample size, opportunistic sampling, and genus-level taxonomic resolution, this work contributes foundational data on vaginal microbiome composition in Mexican pregnancies, establishes stable feature associations warranting replication, and delineates a principled pathway toward future externally validated PTB prediction models that could inform precision medicine approaches in Latin American populations.

The path forward requires multi-center validation cohorts with standardized longitudinal sampling, integration of established clinical predictors (cervical length, obstetric history), species-level microbiome profiling, mechanistic multi-omics studies, and ultimately, intervention trials testing whether microbiome-targeted therapies reduce preterm birth incidence. Until these milestones are achieved, microbiome-based PTB screening remains a research question rather than a clinical reality. This study takes one transparent, methodologically rigorous step along that long translational pathway.

## Data Availability

Analysis code, including nested cross-validation workflows and ANCOM-BC2 implementation, processed microbiome abundance tables, clinical metadata (de-identified), and data dictionaries are available at https://github.com/martinruhle/Mexican-PretermBirth-analysis. Raw 16S rRNA gene sequencing data have been deposited in NCBI's Sequence Read Archive (SRA) under BioProject accession PRJNA1440471.
