# Supplementary material for: Leakage-aware machine learning reveals structured clinical and vaginal microbiome patterns associated with preterm birth in a Mexican cohort
Source: Front Glob Womens Health. 2026 May 7;7:1799518. doi: 10.3389/fgwh.2026.1799518 (PMC13190461; doi:10.3389/fgwh.2026.1799518)
Supplement: Supplementary file 1 [file supplementaryfile1.pdf]

Supplementary Material

1 SUPPLEMENTARY TABLES

Table S1. Performance of all 12 primary model configurations

Algorithm	Features	Microbiome	AUROC	PRAUC	Sensitivity	Specificity
Random Forest	Data-driven	Full	0.813 ± 0.110	0.592 ± 0.209	0.600 ± 0.365	0.613 ± 0.289
Random Forest	Data-driven	ANCOM	0.791 ± 0.170	0.600 ± 0.208	0.533 ± 0.380	0.553 ± 0.299
Elastic Net	Literature	ANCOM	0.789 ± 0.190	0.803 ± 0.210	0.200 ± 0.298	0.493 ± 0.325
Elastic Net	Data-driven	ANCOM	0.784 ± 0.067	0.566 ± 0.165	0.600 ± 0.365	0.653 ± 0.238
Elastic Net	Literature	Full	0.760 ± 0.117	0.814 ± 0.171	0.200 ± 0.298	0.627 ± 0.293
Elastic Net	DREAM	ANCOM	0.736 ± 0.208	0.794 ± 0.192	0.267 ± 0.279	0.660 ± 0.369
Random Forest	Literature	Full	0.727 ± 0.133	0.622 ± 0.171	0.267 ± 0.279	0.827 ± 0.119
Random Forest	DREAM	ANCOM	0.711 ± 0.138	0.677 ± 0.186	0.567 ± 0.279	0.287 ± 0.252
Elastic Net	DREAM	Full	0.684 ± 0.206	0.809 ± 0.151	0.200 ± 0.183	0.633 ± 0.506
Random Forest	Literature	ANCOM	0.662 ± 0.075	0.688 ± 0.130	0.533 ± 0.447	0.493 ± 0.325
Random Forest	DREAM	Full	0.627 ± 0.128	0.662 ± 0.159	0.800 ± 0.183	0.167 ± 0.167
Elastic Net	Data-driven	Full	0.604 ± 0.182	0.632 ± 0.135	0.200 ± 0.183	0.860 ± 0.142

Table S2. Per-fold performance metrics for the best model (Random Forest, Data-driven features, Full microbiome) used in the permutation test. Each fold corresponds to a test set from the nested cross-validation. Metrics include the optimal threshold determined within each fold, AUROC, PRAUC, Sensitivity, Specificity, and Balanced Accuracy.

Fold	n_test	n_ptb	threshold	AUROC	PRAUC	Sensitivity	Specificity	Balanced Accuracy
1	9	3	0.219	0.778	0.511	1.000	0.500	0.750
2	9	3	0.200	0.778	0.502	0.667	1.000	0.833
3	9	3	0.253	0.944	0.454	0.667	0.833	0.750
4	9	3	0.230	0.667	0.532	0.667	0.333	0.500
5	7	2	0.204	0.900	0.963	0.000	0.400	0.200

Table S3. ANCOM-BC2 differentially abundant taxa selected per outer cross-validation fold ($p < 0.10$). Taxa consistently selected across all five folds are indicated in bold. Selection was performed independently within each fold to prevent data leakage.

Fold	Taxa Selected	Count
1	*f__Pleomorphomonadaceae*, ***Mycoplasma***, ***Peptostreptococcus***	3
2	*Escherichia–Shigella*, *f__Comamonadaceae*, *f__Rhizobiaceae*, *Finegoldia*, *Mobiluncus*, *Murdochiella*, ***Mycoplasma***, *Peptoniphilus*, ***Peptostreptococcus***	9
3	*Enhydrobacter*, *f__Pleomorphomonadaceae*, *Gordonia*, ***Mycoplasma***, *o__Saccharimonadales*, ***Peptostreptococcus***, *Propionibacterium*, *Streptococcus*	8
4	*Bifidobacterium*, *Bosea*, *f__Rhizobiaceae*, *Kocuria*, ***Mycoplasma***, ***Peptostreptococcus***	6
5	*f__Bifidobacteriaceae*, *f__Comamonadaceae*, ***Mycoplasma***, ***Peptostreptococcus***	4
**Mean ± SD**		6.0 ± 2.5
**Range**		3–9

Sensitivity Analysis 1: Exclusion of the 20-Week Pregnancy Loss Case

To address the potential outsized influence of a single extreme observation, we performed a sensitivity analysis excluding the participant who delivered at exactly 20.0 weeks' gestation. This case is a clear outlier relative to the PTB group mean (33.7 weeks) and represents a clinical gray zone between spontaneous abortion and preterm birth. Following exclusion (revised cohort: n = 42 subjects, 13 PTB cases), all 12 model configurations were re-evaluated. Overall discrimination was preserved, with ANCOM-based models consistently improving. This confirms that the key findings and the structural clinical-microbiome patterns identified in the primary analysis are not artificially driven by this single borderline observation.

Table S4. Performance Excluding the 20-Week Case

Algorithm	Features	Microbiome	AUROC	PRAUC	Sensitivity	Specificity
Elastic Net	Data-driven	ANCOM	0.860 ± 0.089	0.739 ± 0.257	0.433 ± 0.435	0.513 ± 0.152
Random Forest	Data-driven	ANCOM	0.858 ± 0.109	0.690 ± 0.267	0.467 ± 0.447	0.687 ± 0.344
Elastic Net	Literature	ANCOM	0.806 ± 0.182	0.863 ± 0.183	0.267 ± 0.365	0.487 ± 0.326
Elastic Net	Literature	Full	0.793 ± 0.209	0.870 ± 0.153	0.133 ± 0.298	0.527 ± 0.265
Elastic Net	DREAM	ANCOM	0.778 ± 0.136	0.817 ± 0.190	0.600 ± 0.548	0.253 ± 0.256
Random Forest	Data-driven	Full	0.742 ± 0.105	0.644 ± 0.212	0.600 ± 0.548	0.553 ± 0.218
Random Forest	DREAM	Full	0.712 ± 0.195	0.698 ± 0.217	0.500 ± 0.373	0.613 ± 0.373
Elastic Net	DREAM	Full	0.707 ± 0.247	0.803 ± 0.178	0.133 ± 0.298	0.560 ± 0.518
Random Forest	DREAM	ANCOM	0.701 ± 0.177	0.749 ± 0.187	0.367 ± 0.415	0.333 ± 0.354
Random Forest	Literature	Full	0.689 ± 0.122	0.682 ± 0.157	0.467 ± 0.506	0.487 ± 0.402
Random Forest	Literature	ANCOM	0.663 ± 0.184	0.792 ± 0.145	0.467 ± 0.506	0.353 ± 0.348
Elastic Net	Data-driven	Full	0.622 ± 0.234	0.753 ± 0.190	0.267 ± 0.365	0.560 ± 0.179

Sensitivity Analysis 2: Restriction to Samples Collected Before 28 Weeks

The gestational age at the last available sample systematically differed between the PTB and term groups due to preterm delivery truncating longitudinal follow-up. To rule out the possibility that the model's discriminative ability was merely driven by samples taken chronologically closer to delivery in the PTB group, we performed a sensitivity analysis restricting the dataset strictly to samples collected before 28 weeks of gestation (65 samples from 39 subjects: 12 PTB, 27 term). As shown in Table S3, discriminative performance remained robust (best AUROC 0.840 $\pm$ 0.146). This indicates that the clinical and microbiome signatures associated with PTB are present early in pregnancy and are not solely late-stage artifacts.

Table S5. Performance Restricted to Samples $<$28 Weeks

Algorithm	Features	Microbiome	AUROC	PRAUC	Sensitivity	Specificity
Random Forest	Data-driven	ANCOM	0.840 ± 0.146	0.692 ± 0.255	0.433 ± 0.435	0.527 ± 0.271
Random Forest	Data-driven	Full	0.787 ± 0.140	0.638 ± 0.203	0.467 ± 0.361	0.400 ± 0.374
Random Forest	Literature	Full	0.756 ± 0.128	0.594 ± 0.161	0.467 ± 0.361	0.747 ± 0.284
Random Forest	DREAM	ANCOM	0.749 ± 0.217	0.669 ± 0.207	0.500 ± 0.500	0.513 ± 0.402
Random Forest	Literature	ANCOM	0.722 ± 0.149	0.678 ± 0.212	0.467 ± 0.506	0.773 ± 0.352
Random Forest	DREAM	Full	0.722 ± 0.105	0.628 ± 0.161	0.400 ± 0.418	0.600 ± 0.469
Elastic Net	DREAM	ANCOM	0.702 ± 0.186	0.728 ± 0.205	0.733 ± 0.253	0.227 ± 0.153
Elastic Net	Data-driven	ANCOM	0.679 ± 0.245	0.706 ± 0.193	0.467 ± 0.506	0.560 ± 0.277
Elastic Net	Literature	Full	0.616 ± 0.029	0.701 ± 0.129	0.567 ± 0.365	0.433 ± 0.438
Elastic Net	DREAM	Full	0.607 ± 0.079	0.667 ± 0.115	0.567 ± 0.365	0.393 ± 0.379
Elastic Net	Data-driven	Full	0.551 ± 0.050	0.605 ± 0.015	0.600 ± 0.418	0.520 ± 0.460
Elastic Net	Literature	ANCOM	0.542 ± 0.134	0.726 ± 0.121	0.667 ± 0.204	0.473 ± 0.271

Sensitivity Analysis 3: Elastic Net Regularization Parameter (λ)

In the primary analysis, Elastic Net models were trained using a fixed, moderate regularization parameter ($\lambda = 0.01$) to prevent overfitting without requiring nested hyperparameter tuning. To ensure results were robust, we conducted a sensitivity analysis testing alternative penalty values ($\lambda \in \{0.001, 0.1\}$). Reducing the penalty to $\lambda = 0.001$ yielded stable and comparable performance (Table S4). Conversely, increasing the penalty to $\lambda = 0.1$ resulted in excessive L1/L2 regularization, shrinking all coefficients to exactly zero and causing complete model collapse (e.g., throwing empty prediction vectors during validation folds). This confirms that $\lambda = 0.01$ provided an appropriate balance of regularization for this dataset.

Table S6. Elastic Net Performance with $\lambda = 0.001$

Algorithm	Features	Microbiome	AUROC	PRAUC	Sensitivity	Specificity
Elastic Net	Literature	ANCOM	0.767 ± 0.182	0.803 ± 0.202	0.333 ± 0.236	0.460 ± 0.333
Elastic Net	Data-driven	ANCOM	0.747 ± 0.205	0.543 ± 0.132	0.633 ± 0.247	0.720 ± 0.198
Elastic Net	DREAM	ANCOM	0.736 ± 0.208	0.794 ± 0.192	0.267 ± 0.279	0.587 ± 0.344
Elastic Net	DREAM	Full	0.709 ± 0.146	0.715 ± 0.176	0.400 ± 0.365	0.693 ± 0.395
Elastic Net	Literature	Full	0.696 ± 0.115	0.757 ± 0.157	0.200 ± 0.298	0.687 ± 0.251
Elastic Net	Data-driven	Full	0.593 ± 0.217	0.625 ± 0.146	0.400 ± 0.365	0.660 ± 0.388

Sensitivity Analysis 4: CLR pseudocount

Table S7. Sensitivity analysis of CLR pseudocount on model discrimination. AUROC (mean ± SD across 5 outer cross-validation folds) for all 12 model configurations under three pseudocount values. The primary analysis used pseudocount $= 0.65$ (decontaminated dataset). Models are sorted by AUROC at the primary pseudocount. $|\Delta|_{\mathrm{max}}$: absolute AUROC difference between pseudocounts 0.5 and 1.0. ANCOM_Taxa configurations are largely invariant to pseudocount because ANCOM-BC2 performs its own internal zero-handling.

**Algorithm**	**Feature sel.**	**Microbiome**	**PC = 0.5** AUROC	**PC = 0.65**[†] AUROC	**PC = 1.0** AUROC	$\|\Delta\|_{\mathrm{max}}$
Random Forest	Data-driven	Full	0.802 ± 0.117	**0.813 ± 0.110**	0.813 ± 0.110	0.011
Random Forest	Data-driven	ANCOM	0.791 ± 0.170	0.791 ± 0.170	0.791 ± 0.170	0.000
Elastic Net	Literature	ANCOM	0.789 ± 0.190	0.789 ± 0.190	0.789 ± 0.190	0.000
Elastic Net	Data-driven	ANCOM	0.784 ± 0.067	0.784 ± 0.067	0.784 ± 0.067	0.000
Elastic Net	Literature	Full	0.771 ± 0.128	0.760 ± 0.117	0.760 ± 0.117	0.011
Elastic Net	DREAM	ANCOM	0.736 ± 0.208	0.736 ± 0.208	0.736 ± 0.208	0.000
Random Forest	Literature	Full	0.716 ± 0.152	0.727 ± 0.133	0.727 ± 0.133	0.011
Random Forest	DREAM	ANCOM	0.711 ± 0.138	0.711 ± 0.138	0.700 ± 0.145	0.011
Elastic Net	DREAM	Full	0.684 ± 0.206	0.684 ± 0.206	0.664 ± 0.209	0.020
Random Forest	Literature	ANCOM	0.662 ± 0.075	0.662 ± 0.075	0.662 ± 0.075	0.000
Random Forest	DREAM	Full	0.649 ± 0.108	0.627 ± 0.128	0.627 ± 0.128	0.022
Elastic Net	Data-driven	Full	0.516 ± 0.188	0.604 ± 0.182	0.571 ± 0.164	0.055

[†] Primary analysis (decontaminated dataset).
Note: fold-level SD (0.067–0.208) substantially exceeds all pseudocount-driven differences.

Sensitivity Analysis 5: Inverse-Frequency Sample Weighting to Address Non-Independence

Within the training folds of our nested cross-validation framework, multiple samples from the same subject are treated as independent observations. Because term subjects contributed an average of 3.0 samples each while PTB subjects contributed only 1.7 samples each, we sought to rule out the possibility that the algorithm's feature selection was artificially skewed toward patterns overrepresented by term subjects with multiple visits.

To test this, we performed a sensitivity analysis implementing *Inverse-Frequency Sample Weighting*. We retained all 110 longitudinal samples but applied case weights during the Random Forest model training. Specifically, each sample was assigned a weight equal to $1/n_{samples}$ for that specific subject. This approach completely neutralizes the bias of unequal sampling depth by ensuring that every subject contributes exactly a total weight of 1.0 to the model, without discarding valuable longitudinal snapshots.

The results demonstrated that our primary model is highly robust to this sampling imbalance:

- **Primary Model (Unweighted):** AUROC = 0.813 $\pm$ 0.110 — PRAUC = 0.592 $\pm$ 0.209
- **Sensitivity Model (Inverse-Frequency Weights):** AUROC = 0.789 $\pm$ 0.186 — PRAUC = 0.655 $\pm$ 0.230

While this sensitivity analysis confirms the robustness of our findings, we decided to report the unweighted, sample-level strategy as our primary methodological approach in the main manuscript for three crucial reasons:

1. **Data Leakage Prevention:** Our nested cross-validation rigidly partitions data at the *subject level*. The model is never tested on a sample from a subject it saw during training, strictly preventing the performance inflation typically associated with non-independent longitudinal samples.
2. **Biological Temporal Dynamics:** The vaginal microbiome is highly dynamic across gestation. By allowing the algorithm to learn from independent temporal snapshots without artificially down-weighting them, we capture the natural progression of dysbiosis that may precede preterm labor, which is biologically more informative than forcing an equalized mathematical representation.
3. **Clinical Translation:** In real-world clinical practice, a physician evaluates a single, point-in-time vaginal swab (not a weighted historical average of a patient's visits). Training the algorithm to classify individual cross-sectional snapshots aligns perfectly with this intended clinical screening use case.

2 SUPPLEMENTARY FIGURES

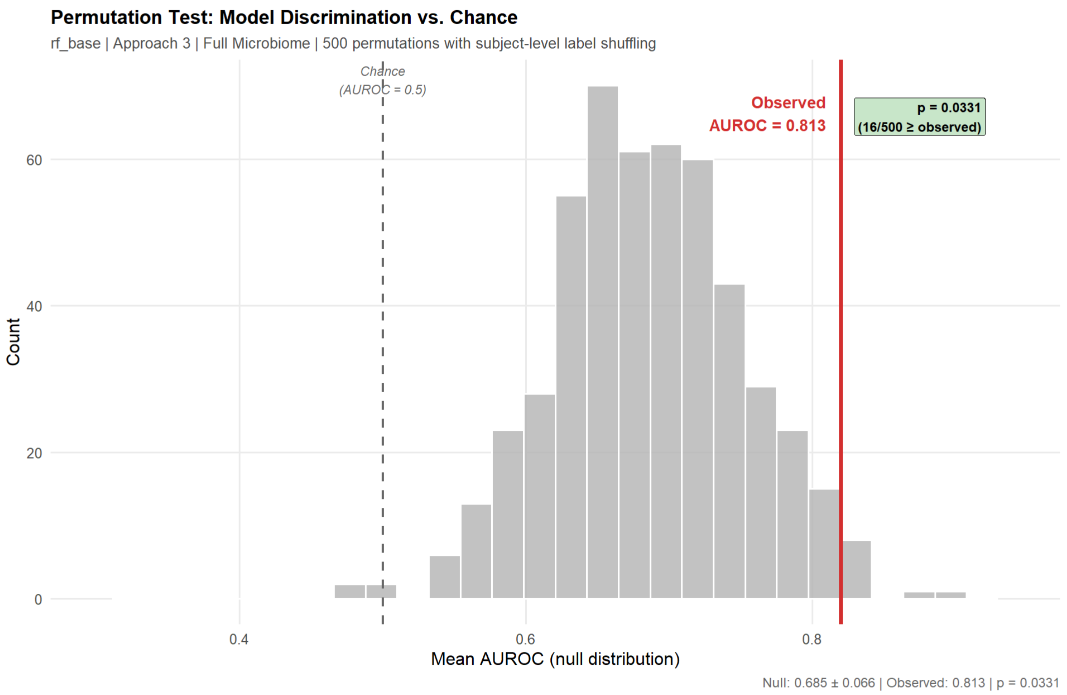

Figure S1. Permutation test results. Distribution of AUROC values under 500 permutations of the outcome labels (shuffled at subject level) through the complete nested cross-validation pipeline. The vertical red line indicates the observed AUROC (0.813) for the best model on the decontaminated dataset. The observed value exceeds 484 out of 500 permuted values, yielding a permutation p-value of 0.0331. The null distribution has mean AUROC = 0.685 $\pm$ 0.066, confirming that the model's discrimination significantly exceeds chance.
